# Outlier Detection in Functional Data Using Adjusted Outlyingness

**DOI:** 10.3390/e28020233

**Published:** 2026-02-16

**Authors:** Zhenghui Feng, Xiaodan Hong, Yingxing Li, Xiaofei Song, Ketao Zhang

**Affiliations:** 1 School of Science, Harbin Institute of Technology, Shenzhen 518055, China; 2 School of Economics, Xiamen University, Xiamen 361005, China; 3 School of Business, Sun Yat-Sen University, Guangzhou 510006, China

**Keywords:** information, non-Gaussian, outlier detection, Mahalanobis distance, functional data, directional outlyingness

## Abstract

In signal processing and information analysis, the detection and identification of anomalies present in signals constitute a critical research focus. Accurately discerning these deviations using probabilistic, statistical, and information-theoretic methods is essential for ensuring data integrity and supporting reliable downstream analysis. Outlier detection in functional data aims to identify curves or trajectories that deviate significantly from the dominant pattern—a process vital for data cleaning and the discovery of anomalous events. This task is challenging due to the intrinsic infinite dimensionality of functional data, where outliers often appear as subtle shape deformations that are difficult to detect. Moving beyond conventional approaches that discretize curves into multivariate vectors, we introduce a novel framework that projects functional data into a low-dimensional space of meaningful features. This is achieved via a tailored weighting scheme designed to preserve essential curve variations. We then incorporate the Mahalanobis distance to detect directional outlyingness under non-Gaussian assumptions through a robustified bootstrap resampling method with data-driven threshold determination. Simulation studies validated its superior performance, demonstrating higher true positive and lower false positive rates across diverse anomaly types, including magnitude, shape-isolated, shape-persistent, and mixed outliers. The practical utility of our approach was further confirmed through applications in environmental monitoring using seawater spectral data, character trajectory analysis, and population data underscoring its cross-domain versatility.

## 1. Introduction

In signal processing and information analysis, the detection and identification of anomalies present in signals constitute a critical research focus. Accurately discerning these deviations is essential for ensuring data integrity and supporting reliable downstream analysis. This task is fundamentally about quantifying and interpreting uncertainty and unexpectedness in data, a domain where information-theoretic concepts like entropy and Kullback–Leibler divergence provide a natural and powerful framework. Entropy, as a measure of uncertainty or information content and mutual information which captures shared information between variables, offers principled methods to distinguish abnormal patterns that deviate from the expected information structure of a signal. Extending this reasoning to the analysis of continuous, high-dimensional signals leads directly to the domain of Functional Data Analysis (FDA). FDA has emerged as a crucial statistical framework for analyzing data where each sample is a function or a continuous curve, observed over a continuum such as time or wavelength [1,2,3,4]. Unlike multivariate data, which consist of vectors in a finite-dimensional Euclidean space, functional data are intrinsically infinite-dimensional objects. This fundamental distinction, treating a curve as a single entity rather than a discrete set of points, allows FDA to preserve and model the inherent smoothness and dynamic structure of the data. However, this very characteristic renders standard multivariate analytical techniques inadequate. In particular, the task of outlier detection, which is a cornerstone of measurement data quality assurance, faces significant challenges when applied to functional data. In FDA, the detection of outliers (observations with abnormal patterns or deviations) is a critical step in measurement data processing. It serves to ensure the accuracy and reliability of subsequent analysis by preventing distorted functional patterns and biased estimates, while also aiming to identify genuine unusual events that may be of primary interest.

**Figure 1:**
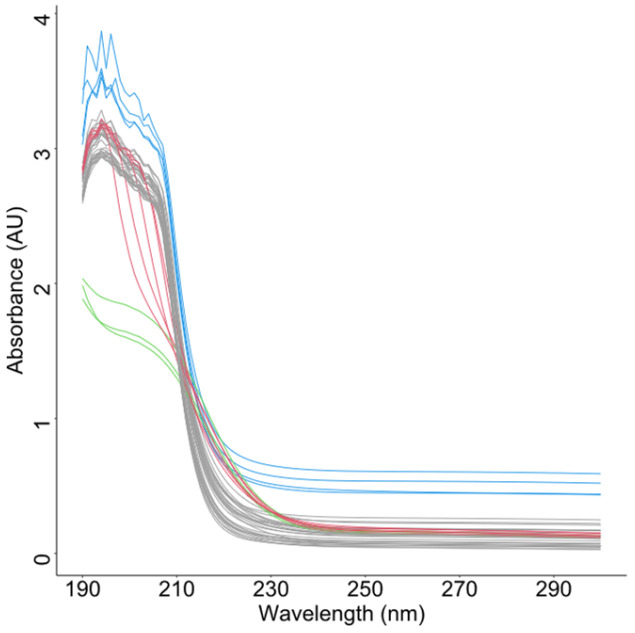
Seawater spectral data from the Xiamen coastal waters in China.

We used seawater spectral absorption data from the Xiamen coastal area, China (Figure 1) to illustrate the critical need for functional outlier detection in practical analysis. Figure 1 displays the seawater spectral absorption curves of 69 samples, classified by color based on distinct spectral shapes: gray (majority), green (magnitude outlier), red (shape-based anomaly), and blue (mean shift). The green, red, and blue curves were designated as outliers due to environmental discontinuity, as they originated from a freshwater environment or an area with potential industrial contamination, unlike the core marine data. The presence of such outliers that affect the magnitude, shape, or both severely distorts functional means, trend estimation, and model parameters. For instance, a shift in magnitude can bias estimates of central tendency, while a change in the shape of a distribution can invalidate assumptions about trends. Therefore, reliably identifying these diverse anomalies is essential before functional modeling is performed.

While recent advances in outlier detection predominantly address multivariate data and streaming data [5,6,7,8], these methods are often inapplicable to functional data. Typically based on classification principles, they struggle with functional data where each sample is a curve originating from an infinite-dimensional function space. Simply discretizing curves fails to capture their intrinsic continuity and structure, creating a significant gap in measurement science for robust functional data analysis. Furthermore, many approaches rely on supervised or clustering techniques [5,9,10,11] that require labeled data. While some recent reviews [5,6] discuss anomaly detection in specific contexts like the IoT, they prioritize industrial applications over the theoretical underpinnings of functional data. Conventional outlier detection methods designed for multivariate data often fail to effectively detect shape-related or dynamic anomalies unique to functional observations. This methodological gap underscores the pressing need for novel approaches specifically tailored to the unique nature of functional data. Other related works include online detection [11], and neural network-based anomaly detection methods [12,13,14,15,16]. Most of them are designed for time series data, relying on strong assumptions of temporal ordering and dependency (e.g., sliding window segmentation and Kalman filtering). As such, they fall outside the scope of this study, which focuses on a formal inference framework for functional data without such constraints.

The complex nature of functional outliers, which can manifest as magnitude shifts, shape deformations, or localized disturbances, demands sophisticated methods capable of capturing diverse anomalous patterns. Although research studies on functional outlier detection are growing [17,18,19,20], they often fail to handle different types of anomalies simultaneously. What is more, a significant limitation persists: many current approaches rely on restrictive distributional assumptions (e.g., Gaussian process) or specific outlier models to ensure theoretical tractability. This reliance limits their practical applicability in real-world scenarios where data distributions are often unknown, complex, or non-Gaussian, which potentially leads to missed detections (false negatives) or misidentifications of valid data points (false positives). This gap underscores the urgent need for flexible and robust detection methods capable of handling diverse functional data types without stringent distributional constraints.

Our work in this paper addressed these gaps by proposing an unsupervised method that leverages the data’s underlying distribution. By utilizing quantiles of a robust measure, our approach relaxes restrictive assumptions like the Gaussian process, offering a novel solution for functional outlier detection. We propose a method to conduct outlier detection for functional data. The detection framework is established by incorporating a novel adjusted outlyingness metric, the robust Mahalanobis distance, and a bootstrap technique. The adjusted outlyingness metric integrates adaptive magnitude outlyingness, scaled variability, and directional skewness to comprehensively characterize diverse anomaly types in functional data. The robust Mahalanobis distance and bootstrap technique help determine the anomaly scores and the detection threshold.

Our method differs from the current reference in the following aspects. First, the literature on outlier detection is extensive; it predominantly focuses on multivariate data, often leveraging clustering-based methodologies [5,6,7,8,9,10,11], while our method is designed for functional data and is not clustering or classification based. Second, in the FDA area, some works make use of metrics like ranks or depths [21] and determine the outliers by hypothesis testing [22,23]. However, they need the assumption of a certain distribution. The distribution assumption is strong in applications. Our method is a distributionally adaptive framework through the systematic integration of the robust Mahalanobis distance with bootstrap resampling. Third, compared with the directional outlyingness metric used in [22] and other metrics in [19], we propose a new metric termed the skewness of directional outlyingness. In addition, we introduce the adjusted directional outlyingness that incorporates data-driven weights to effectively capture local features.

This paper makes several contributions. First, it addresses the gap in outlier detection for functional data, a domain underserved by methods designed for multivariate data. Methodologically, we propose a new metric termed the skewness of directional outlyingness, together with the weighted directional outlyingness metrics that provide a more nuanced characterization of anomalies. Second, we introduce a distributionally robust framework that innovatively integrates bootstrap resampling for threshold estimation, thereby eliminating the reliance on restrictive Gaussian process assumptions. The synergy of these elements results in a flexible, unsupervised algorithm that enhances the state-of-the-art in functional data quality assurance and is capable of detecting diverse types of anomalies.

The remainder of this paper is structured as follows: In Section 2, we introduce the framework and detection methodology. In Section 3, we validate the approach through simulation studies that compare performance against established methods. In Section 4, we demonstrate practical applications. We present concluding remarks and research implications in Section 5.

## 2. Methodology

Functional data outliers are classified into two types: magnitude anomalies (curves displaced vertically from the functional envelope) and shape anomalies (curves within the majority envelope but exhibiting a different trend) [24]. Magnitude outliers are easier to detect than shape outliers through the visual analysis of the samples. Based on the extent to which anomalies occur in the sample, functional data outliers can also be categorized into isolated anomalies and persistent anomalies [25]. Persistent anomalies can be further subdivided into shift outliers, magnitude outliers, and shape outliers. In practice, isolated outliers and magnitude outliers are considered as a type of shape outlier. In this paper, we focus on all these types of outliers.

We consider a stochastic process $X\left(t\right):I\to R^{p}$ that maps from a compact set I to $R^{p}$ according to the probability function $F_{X\left(t\right)}$. With t in I, $X\left(t\right)={\left(X^{1}\left(t\right),X^{2}\left(t\right),\ldots ,X^{p}\left(t\right)\right)}^{T}$ is a p-dimensional function of t. $F_{X\left(t\right)}$ denotes the distribution of $X\left(t\right)$ at each time point t. In Figure 1, for p=1, each absorption spectrum constitutes a functional observation, where $t\in \left[190,\;300\right]$ nm denotes a wavelength, with measurements recorded at 1 nm intervals. Functional data lack a natural ordering, which necessitates comprehensive statistical measures for curve comparison. To sort functional data, a comprehensive measure that evaluates the entire function is required. To address this, we define the adjusted outlyingness vector, which encodes the magnitude, scale, and shape characteristics of each functional observation. This transformation reduces the functional outlier detection problem to multivariate anomaly identification in the adjusted outlyingness space. Building on this, we propose the adjusted outlyingness detection (AOD) method.

### 2.1. Adjusted Outlyingness

To propose the adjusted outlyingness indices, we propose two weights and introduce the definition of directional outlyingness.

**Definition 1.** 
*(Weight *$w_{1}$*). For the stochastic process *$X\left(t\right)$*, at time *t*, the weight *$w_{1}\left(t\right)\in R^{p}$* is*

(1) $${\left(w_{1}\left(t\right)\right)}_{j}=w_{1}^{j}\left(t\right)=\frac{sd\left(X^{j}\left(t\right)\right)}{\int_{I}sd\left\{X^{j}\left(t\right)\right\}dt},j=1,\ldots ,p$$

* where* $sd\left(X^{j}\left(t\right)\right)$ *is the standard deviation of function component* $X^{j}\left(t\right)$ *at time* t*.*

**Remark 1.**  $w_{1}\left(t\right)$ *can be estimated from the standard deviations of sample values across different curves at the observed time points within the dataset. It quantifies the degree of variation at* t*. Low variability indicates curve consensus and reduced anomaly potential. High variability reflects dispersion and elevated outlier likelihood. Although* $w_{1}\left(t\right)$ *was studied in [26,27], its application to outlyingness remains unexplored.*

**Definition 2.** 
 *(Weight* $w_{2}$*)*

(2) $${\left(w_{2}\left(t\right)\right)}_{j}=w_{2}^{j}\left(t\right)=\frac{sd\left\{{\left(X^{j}\right)}^{\prime }\left(t\right)\right\}}{\int_{I}sd\left\{{\left(X^{j}\right)}^{\prime }\left(t\right)\right\}dt},j=1,\ldots ,p$$

*where* ${\left(X^{j}\right)}^{\prime }\left(t\right)=\frac{dX^{j}\left(t\right)}{dt},\;\;j=1,\ldots ,p$*.*

**Remark 2.** 
*The weight function *$w_{2}(t)$ *is the derivative-based counterpart to* $w_{1}(t)$*.* *It operates on* ${\left(X^{j}\right)}^{\prime }\left(t\right),\;\;j=1,\ldots ,p$*, thereby quantifying variability in the rate of functional change at each t. This generalized framework extends beyond prior weighting schemes [28] by explicitly capturing shape dynamics. An elevated standard deviation in* ${\left(X^{j}\right)}^{\prime }\left(t\right)$ *at any t indicates heightened heterogeneity in instantaneous shape variation across curves. Such dispersion signals potentially shape anomalies localized at t, which warrants increased sensitivity to functional behavior in these critical regions.*

**Remark 3.** 
*The two proposed weight functions, *$w_{1}\left(t\right)$ *and* $w_{2}(t)$ *, capture distinct structural features of the functional data. The weight* $w_{1}\left(t\right)$ *, derived from the standard deviation, quantifies the pointwise variability of the process. It assigns higher importance to regions with greater dispersion, making it robust and effective for detecting magnitude-driven outliers. However, it may overlook subtle shape anomalies in regions with low variance. In contrast,* $w_{2}(t)$ *incorporates derivative information, making it highly sensitive to the local rate of change. This allows for the detection of shape-related anomalies, such as sharp transitions or inflection points, even in the absence of high magnitude variability. Consequently, the choice between* $w_{1}\left(t\right)$ *and* $w_{2}(t)$ *, should be guided by the specific nature of the anomalies expected in the application:* $w_{1}\left(t\right)$ *is preferable for magnitude outliers, while* $w_{2}(t)$ *, is superior for identifying shape deviations.*

**Definition 3.** 
*(Directional Outlyingness, DO, [22])*

(3) $$O\left\{X\left(t\right),F_{X\left(t\right)}\right\}=o\left\{X\left(t\right),F_{X\left(t\right)}\right\}\cdot v\left(t\right)$$

*where* $o\left(X\left(t\right),F_{X\left(t\right)}\right)=\frac{\left\|X\left(t\right)-\mu \left(F_{X\left(t\right)}\right)\right\|}{\sigma \left(F_{X\left(t\right)}\right)}$*,* μ *and* σ *are the location and scale measures of* $X\left(t\right)$*, respectively.* $\left\|\cdot \right\|$ *represents the norm of a vector.* $v\left(t\right)=\left\{X\left(t\right)-median\left(X\left(t\right)\right)\right\}/\left\|X\left(t\right)-median\left(X\left(t\right)\right)\right\|$ *is the spatial sign [21,29].*

DO not only measures the eccentricity of the curve at time point t but also captures the direction $v\left(t\right)$ of its eccentricity. It characterizes the deviation of a multivariate observation from the central tendency of a distribution by jointly accounting for the magnitude and the direction of the deviation. Unlike traditional scalar measures of outlyingness or data depth, which quantify only the extent of atypicality, directional outlyingness retains directional information with respect to the data center. It provides when evaluated a local description of how a curve departs from the central behavior at each time point. For details, please refer to [22].

With these notations and definitions, we propose the following adjusted outlyingness indices.

**Definition 4.** 
*(Adjusted Mean of directional Outlyingness, AMO) For weights *$w_{1}$ *and* $w_{2}$ *, two types of weighted means are defined as*

(4) $${AMO}_{1}\left(X,F_{X}\right)=\int_{I}O(X\left(t\right),F_{X\left(t\right)})⊙w_{1}\left(t\right)dt$$

(5) $${AMO}_{2}\left(X,F_{X}\right)=\int_{I}O(X\left(t\right),F_{X\left(t\right)})⊙w_{2}\left(t\right)dt$$

*where ⊙ represents the Hadamard (element-wise) product operator. Consequently,* ${AMO}_{1}$ *and* ${AMO}_{2}$ *are* p*-dimensional vectors.*

**Definition 5.** 
*(Adjusted Variance of directional Outlyingness, AVO) With weights *$w_{1}$ *and* $w_{2}$ *, two types of weighted variances are defined as*

(6) $$AVO_{1}\left(X,F_{X}\right)=\sum_{j=1}^{p}\int_{J}{\left(O\left(X^{j}\left(t\right),F_{X\left(t\right)}\right)-{AMO}_{1}\left(X^{j},F_{X}\right)\right)}^{2}{\left(w_{1}\left(t\right)\right)}_{j}dt$$

(7) $$AVO_{2}\left(X,F_{X}\right)=\sum_{j=1}^{p}\int_{J}{\left(O\left(X^{j}\left(t\right),F_{X\left(t\right)}\right)-{AMO}_{2}\left(X^{j},F_{X}\right)\right)}^{2}{\left(w_{2}\left(t\right)\right)}_{j}dt$$

**Remark 4.**  ${AMO}_{k}\left(X,F_{X}\right)$ *and* $AVO_{k}\left(X,F_{X}\right)$*,* k=1, 2, *reduce to Mean directional Outlyingness (MO) and Variation of directional Outlyingness (VO) [22], respectively, if and only if the weight function satisfies* $w_{k}\left(t\right)=\lambda {\left(J\right)}^{-1}1,\;$*where* $1={\left(1,\ldots ,1\right)}^{T}$*. For the univariate case (*p=1*), the Hadamard product ⊙ simplifies to scalar multiplication. Hence,* ${AMO}_{k}\left(X,F_{X}\right)=\int_{I}O\left\{X\left(t\right),F_{X\left(t\right)}\right\}w_{k}\left(t\right)dt$*, and* $AVO_{k}\left(X,F_{X}\right)=\int_{J}{\left(O\left(X\left(t\right),F_{X\left(t\right)}\right)-{AMO}_{1}\left(X,F_{X}\right)\right)}^{2}w_{k}\left(t\right)dt,k=1,2.$ *The properties of the indices* ${AMO}_{k}$ *and* $AVO_{k}$ *are inherently determined by the chosen weight function*$\;w_{k}\left(t\right)$*,* k=1,2*. The indices based on*${\;w}_{1}\left(t\right)$ *(*${AMO}_{1}$ *and* $AVO_{1}$*) prioritize magnitude-driven variability. They provide a robust measure of overall outlyingness and are ideal for detecting outliers in data with smooth functional shapes where dispersion is the key feature. However, they may overlook subtle shape anomalies in low-variance regions. Conversely, the indices based on w_2 (t) (*${AMO}_{2}\;$*and* $AVO_{2}$*) leverage derivative information to detect shape-related outliers, such as abrupt shifts or changes in slope. While* ${AMO}_{2}$ *and* $AVO_{2}$ *excel at identifying structural irregularities that magnitude-based measures miss; they can be sensitive to high-frequency noise. Therefore, the choice between the two weighting schemes should be guided by whether magnitude deviations or shape dynamics are of primary interest in the data.*

**Definition 6.**  *(Skewness of directional Outlyingness, SO)*

(8) $$SO\left(X,F_{X}\right)=\frac{1}{\lambda \left(J\right)}\sum_{j=1}^{p}\int_{J}{\left[\frac{\left[O\left(X^{j}\left(t\right),F_{X\left(t\right)}\right)-MO\left(X^{j},F_{X}\right)\right]}{\sqrt{VO\left(X^{j},F_{X}\right)}}\right]}^{3}dt$$

*where* $MO\left(X,F_{X}\right)=\int_{I}O\left\{X\left(t\right),F_{X\left(t\right)}\right\}w\left(t\right)dt$*, *$VO\left(X,F_{X}\right)=\int_{I}{\left\|O\left(X\left(t\right),F_{X\left(t\right)}\right)-MO\left(X,F_{X}\right)\right\|}^{2}w\left(t\right)dt$ *in [22].*

**Remark 5.**  SO *quantifies positional and shape deviations by assessing asymmetry relative to the functional mean. As a novel third-order moment metric,* SO *complements existing measures (*${AMO}_{1}$*,* ${AMO}_{2}$*,* $AVO_{1}$*, and*$\;AVO_{2}$*) by capturing distributional asymmetry in directional outlyingness. Specifically, SO identifies both the magnitude and direction (positive/negative) of skewness, which provides critical insights into asymmetric shape characteristics undetectable through lower-order moments.*

The proposed $AMO_{1}$ metric advances beyond the mean directional outlyingness through adaptive weighting. A key limitation of mean directional outlyingness uniform weighting is that symmetrically opposing deviations can cancel out, obscuring the true anomaly magnitude. In contrast, $AMO_{1}$ strategically emphasizes regions that exhibit significant shape variation. This weighting enhances sensitivity to partial anomalies while preserving location measurement integrity. Concurrently, $AVO_{2}$ extends the variation of directional outlyingness by amplifying shape divergence signals. While the variation of directional outlyingness quantifies general dispersion, $AVO_{2}$ specifically magnifies localized shape anomalies. This ensures that outliers manifest pronounced statistical signatures at the affected time points.

Integrating these advances with the skewness metric *SO*, we define the adjusted outlyingness (AO) vector $AO=\left(AMO_{1},AVO_{2},SO\right)$. This triple metric representation comprehensively encodes functional curve characteristics (position, shape, and asymmetry) while achieving critical dimensionality reduction from the original infinite-dimensional space.

### 2.2. Adjusted Outlyingness Detection Method

The core innovation of our approach lies in the transformation of functional outlier detection into a multivariate problem through the AO vector $AO=\left(AMO_{1},AVO_{2},SO\right)$ and the bootstrap estimation of its distribution. This representation distills the essential features of functional curves while achieving significant dimensionality reduction. Subsequent outlier identification operates within this AO space using robust Mahalanobis distance metrics. For notation convenience, $\left(AMO_{1},AVO_{2},SO\right)$ is denoted by $\left(AMO,AVO,SO\right)$. To address the critical limitation of the conventional Mahalanobis distance, we introduce the robust Mahalanobis distance ($\sqrt{BRMD^{2}\;(AO)}$). These elements constitute our proposed AOD framework.

The complete workflow processes functional observations $X_{1},\ldots ,X_{n}$ as follows: Each curve is mapped to its ${AO}_{i}=(AMO_{i},AVO_{i},SO_{i})$ representation, from which $BRMD^{2}\left(AO\right)$ is computed. The α-th quantile of the robust Mahalanobis distance ($BRMD_{\alpha }^{2}$) is then determined via the smoothed bootstrap. Observations that exceed this threshold, $BRMD^{2}\left({AO}_{i}\right)>BRMD_{\alpha }^{2}$, are flagged as outliers. This integrated approach combines the representational efficiency of AO with the robustness of bootstrap-based distance metrics.

#### 2.2.1. Robust Mahalanobis Distance

The Mahalanobis distance [30] is a distance measure that quantifies the distance of a sample from the center:

(9) $$MD\left(Z\right)=\sqrt{{\left(Z-\mu \right)}^{T}\Sigma^{-1}\left(Z-\mu \right)}$$

where $Z\in R^{p},\;p\in N_{+}$, μ is the mean of Z, and Σ is the covariance matrix of Z. When μ and Σ are unknown, they need to be estimated. As the variance estimate used in $MD\left(Z\right)$ is easily influenced by outliers, the robust Mahalanobis distance is required. To obtain the robust Mahalanobis distance $\sqrt{BRMD^{2}(AO)}$, we use two approaches: the bootstrap minimum covariance determinant (MCD) robust Mahalanobis distance statistic or the FAST-MCD [31].

(1) *Bootstrap minimum covariance determinant robust Mahalanobis distance statistic*

We introduce a bootstrap-enhanced MCD estimator for the robust Mahalanobis distance computation. This approach strategically combines bootstrap resampling with MCD principles to mitigate outlier contamination in parameter estimation. The core innovation lies in adaptive subsampling during bootstrap iterations; multiple subsample proportions are evaluated, with the optimal proportion selected by minimizing the determinant of the covariance matrix estimate. This accommodates unknown outlier prevalence in real-world data.

Formally, for each bootstrap iteration b, candidate subsamples yield estimates $\left({\hat{\mu }}_{b},{\hat{\Sigma }}_{b}\right)$. The optimal robust estimators are determined by $\left(\hat{\mu },\hat{\Sigma }\right)=argmin\left\{det\left(\Sigma_{b}(\mu_{b})\;\right)\right\}$. The robust Mahalanobis distance $\sqrt{(BRMD^{2}\left(AO\right)}$ is then defined as the square root of $BRMD^{2}\left(AO\right)$, where

(10) $$BRMD^{2}\left(AO\right)={\left(AO-\hat{\mu }\right)}^{T}{\left(\hat{\Sigma }\right)}^{-1}\left(AO-\hat{\mu }\right)$$

and $\left(\hat{\mu },\hat{\Sigma }\right)$ are defined above. This dual-phase approach, iterative subsample optimization followed by distance computation, effectively insulates estimates from anomalous influences while maximizing information extraction from uncontaminated data regions. The implementation detail is formalized in Algorithm 1.
**Algorithm 1.** Bootstrap Minimum Covariance Determinant Robust Mahalanobis Distance Statistic**Input**: ${AO}_{i},i=1,2\ldots ,n$: The adjusted outlyingness of $X_{i},i=1,\ldots ,n$; percent: The sampling proportions, here $percent={\left[0.85,\;0.9,\;0.95\right]}^{T}$; B: The total resampling rounds **Output**: $BRMD^{2}\left({AO}_{i}\right),i=1,\ldots ,n$: The bootstrap minimum covariance determinant robust Mahalanobis distance statistics **Steps**: (1)**for** b in 1: B
    **for** *j* in 1: length (percent)
        c = $percent\left[j\right]$
    D=Resampling c of whole samples
            Calculate the mean ${\hat{\mu }}_{j}$ and covariance matrix ${\hat{\Sigma }}_{j}$ of D
    **end**
        $\left({\hat{\mu }}_{b},{\hat{\Sigma }}_{b}\right)=argmin\left\{det\left(\Sigma_{j}(\mu_{j})\;\right)\right\}$
      **end**
(2)$\hat{\Sigma }=\underset{b}{mean}\left({\hat{\Sigma }}_{b}\right)$; $\hat{\mu }=\underset{b}{mean}\left({\hat{\mu }}_{b}\right)$(3)Calculate $BRMD^{2}\left({AO}_{i}\right)$ based on $BRMD^{2}\left({AO}_{i}\right)={\left({AO}_{i}-\hat{\mu }\right)}^{T}{\left(\hat{\Sigma }\right)}^{-1}\left({AO}_{i}-\hat{\mu }\right),i=1,\ldots ,n$.

(2) *FAST-MCD*

FAST-MCD [31] can quickly obtain robust estimates, and it is faster and more accurate when the sample size is large. The key step in this process is referred to as the C-step. The C-step procedure iteratively refines the robust location and scatter estimates for a p-dimensional dataset. Beginning with an initial h-sized subset $H_{1}$, it computes the mean ${\hat{\mu }}_{1}$ and covariance ${\hat{\Sigma }}_{1}$. Mahalanobis distances for all observations are then calculated relative to these estimates. The subset is updated to $H_{2}$ by selecting h observations with the smallest distances. This process repeats, thereby generating successively refined estimates (${\hat{\mu }}_{2}$, ${\hat{\Sigma }}_{2}$) with monotonically decreasing covariance determinants. Convergence occurs when either the determinant becomes singular or subsequent determinants stabilize ($\det\left({\hat{\Sigma }}_{2}\right)=\det\left({\hat{\Sigma }}_{1}\right)$), which indicates optimal robust parameter estimation. The algorithm for FAST-MCD is in [31].

#### 2.2.2. Threshold Determination

Outliers are identified by applying the robust Mahalanobis distance statistics, which quantifies the deviation of samples from the multivariate center. To decide whether a sample is considered anomalous, we need to set a threshold. To avoid the assumption of a stationary Gaussian process for the data, we propose using the smoothed bootstrap [32] to determine the threshold, which is applicable to data from non-Gaussian processes.

Sampling probabilities $p={\left(p_{1},\ldots ,p_{n}\right)}^{T}$ in the bootstrap are weighted by the Mahalanobis depth to minimize outlier influence. Depth is computed as

(11) $$BMHD\left({AO}_{i}\right)={\left[1+BRMD^{2}\left({AO}_{i}\right)\right]}^{-1},i=1,\ldots ,n$$

Following the principle of assigning sampling probabilities based on depth measures [33], the sampling probability for each sample in the bootstrap is defined as

(12) $$p_{i}=\frac{BMHD\left({AO}_{i}\right)}{\Sigma_{j=1}^{n}BMHD\left({AO}_{j}\right)},i=1,\ldots ,n$$

Each bootstrap replication b generates smoothed functional data:

(13) $$X_{i}^{sb}\left(t\right)=X_{i}^{b}\left(t\right)+z_{i}^{b}\left(t\right),i=1,\ldots ,n$$

where $z_{i}^{b}\left(t\right)\sim N\left(0,\beta \Sigma_{X}\right)$ and β is the smoothing coefficient, usually set to 0.05 [32]. $\Sigma_{X}$ is the covariance matrix of $X\left(t\right)$. {$X_{1}^{sb}(t),\ldots ,X_{n}^{sb}(t)\}$ is used to obtain their ${AO}_{i}^{b}$, and then $BRMD^{2}\left({AO}_{i}^{b}\right)$. $BRMD_{1-\alpha ,b}^{2}$ is obtained as the $\left(1-\alpha \right)$ quantile of ${\left\{BRMD^{2}\left({AO}_{i}^{b}\right)\right\}}_{i=1}^{n}$. The final threshold is $BRMD_{1-\alpha }^{2}=\underset{b}{mean}\left(BRMD_{1-\alpha ,b}^{2}\right)$ across B times. Samples exceeding this threshold are classified as outliers. Algorithm 2 shows the procedure for AOD.
**Algorithm 2.** Adjusted Outlyingness Detection**Input**: $X_{1}\left(t\right),\ldots ,X_{n}\left(t\right)$: Data set; β: The smoothing coefficient; 1−α: Quantile value; B: The total resampling rounds **Output**: Outlier Detection results (true or false) **Steps**:
(1)Calculate the sample covariance matrix $\Sigma_{X}=cov\left(X\left(t\right)\right)$
(2)Calculate the adjusted outlyingness ${AO}_{i},\;\;i=1,\ldots ,n$ based on Equations (4), (7) and (8)(3)Get $BRMD^{2}\left({AO}_{1}\right),\ldots ,BRMD^{2}\left({AO}_{n}\right)$ according to or FAST-MCD(4)Get the resampling probability $p_{i}$ according to Equation (12)(5)**for** b in 1: B
Resample n samples $X_{1}^{b}\left(t\right),\ldots ,X_{n}^{b}\left(t\right)$ from $X_{1}\left(t\right),\ldots ,X_{n}\left(t\right)$ with replacement according to $p_{i},i=1,\ldots ,n$Let $X_{i}^{sb}\left(t\right)=X_{i}^{b}\left(t\right)+z_{i}^{b}\left(t\right)$, where $z_{i}^{b}\left(t\right)\sim N\left(0,\beta \Sigma_{X}\right)$
Calculate $BRMD^{2}$ of $X_{1}^{sb}\left(t\right),\ldots ,X_{n}^{sb}\left(t\right)$ based on their ${AO}_{i}^{sb}\;$ according to Algorithm 1
Determine the $\left(1-\alpha \right)$-th quantile of the robust Mahalanobis distances, denoted as ${\left(BRMD^{2}\right)}_{1-\alpha }^{b}$
**end**(6)Compute the mean of the B quantile estimates: $BRMD_{1-\alpha }^{2}=\underset{b}{mean}\left[{\left(BRMD^{2}\right)}_{1-\alpha }^{b}\right]$
(7)Output: $\{i:BRMD^{2}\left({AO}_{i}\right)>BRMD_{1-\alpha }^{2}\}.$

## 3. Simulation Study

To demonstrate the effectiveness and practical significance of the skewness metric, we use the following Model 0 for illustration.

Model 0:

Normal samples: $X\left(t\right)=4t+\varepsilon \left(t\right)$, where $\varepsilon \left(t\right)$ is a zero-mean normal distribution, and the covariance function is $\gamma \left(s,t\right)=0.3\exp\left\{-\left|s-t\right|\right\}.$

Isolated shape outlier samples: $X\left(t\right)=4t+2U{\times I}_{\left\{T\leq t\leq T+0.05\right\}}+\varepsilon \left(t\right)$, where U has a 50% probability of being 1 and a 50% probability of being −1; $I_{\left\{T\leq t\leq T+0.05\right\}}$ is an indicator function that equals 1 in the interval $\left[T,\;T+0.05\right]$ and 0 otherwise, with T drawn from a uniform distribution $U\left(0,0.8\right)$.

**Figure 2:**
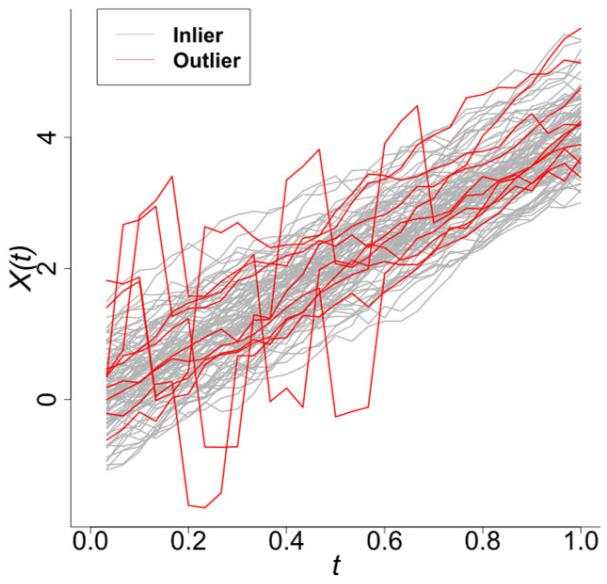
Samples generated from Model 0 (with a total of 100 samples, 30 observation points).

**Figure 3:**
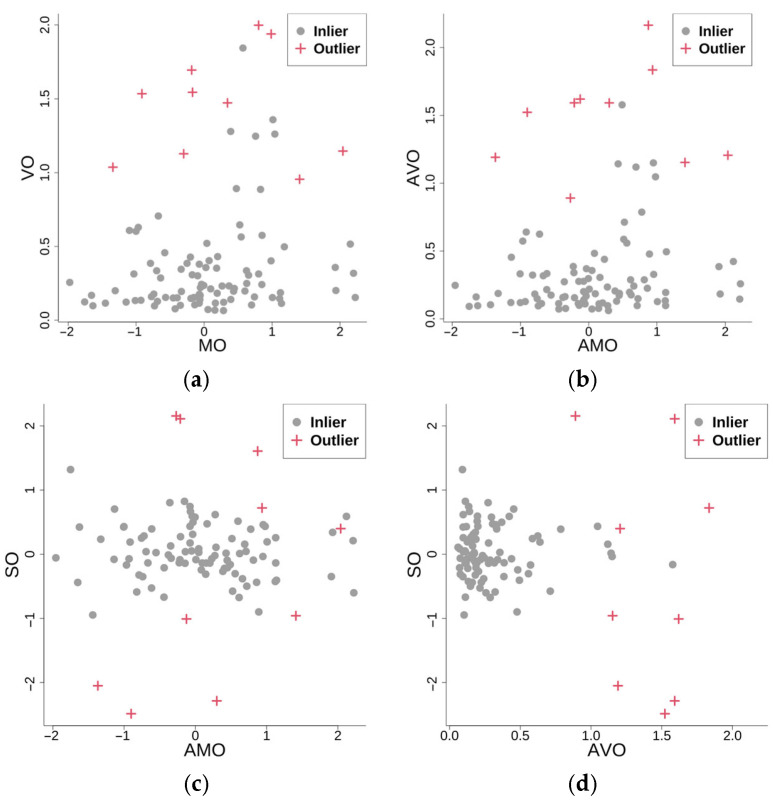
Scatter plots of different outlyingness statistics under Model 0. (**a**) 2D plot of (MO,VO) in [22], (**b**) 2D plot of $\left(AMO,\;AVO\right),$ (**c**) 2D plot of $\left(AMO,\;SO\right),$ (**d**) 2D plot of (AVO, SO).

The simulation comprises n=100 functional samples with k=30 observation points, which contain 10% anomalous curves (red, Figure 2). Figure 3 presents scatterplots of the different outlyingness statistics. The distinct separation between nominal and anomalous samples in the (AMO, AVO), (AVO,SO) planes demonstrates the discriminative power of these metrics. Anomalies consistently occupy peripheral regions beyond the nominal sample clusters, which validates the detection efficacy of the integrated statistics.

In the following simulation study, we evaluated the proposed method through two integrated components: performance assessment on univariate and multivariate functional outlier detection. We set the outlier proportion to c and detection threshold to the α=0.95 quantile. We replicated each simulation 100 times and averaged the metrics. We quantified performance using true positive rate ($p_{c}$, the proportion of correctly identified anomalies), false positive rate ($p_{f}$, the proportion of misclassified nominal samples). Thus, a high $p_{c}$ coupling a low $p_{f}$ indicated superior detection performance. The visual representations consistently depict anomalous samples in red and nominal samples in gray.

### 3.1. Univariate Functional Data

We compared the following methods: W1_1, the proposed AOD method using the bootstrap MCD robust Mahalanobis distance statistic; W1_2, the proposed AOD method using FAST-MCD; W2, the DO detection method [22]; W3, the functional boxplot [34]; W4, the functional bagplot [24]; and W5, FAST-MUOD [19].

We designed four examples: magnitude outliers, persistent shape outliers, isolated shape outliers, and mixed outliers. For each simulation configuration, we used samples with k=30 equidistant observation points over [0,1]. Additionally, to demonstrate the advantages of relaxing the Gaussian distribution assumption required in W2, we further implemented all examples under three error distributions:

(1) Scenario 1: Multivariate Gaussian distribution. The error term $\varepsilon \left(t\right)$ follows a zero-mean normal distribution and the covariance function is $\gamma \left(s,t\right)=0.3\exp\left\{-\left|s-t\right|\right\}$.

(2) Scenario 2: Multivariate t-distribution. The error term $\varepsilon \left(t\right)$ follows a t-distribution with 5 degrees of freedom and the covariance function is $\gamma \left(s,t\right)=0.3\exp\left\{-\left|s-t\right|\right\}$.

(3) Scenario 3: Multivariate skewed t-distribution. The error term $\varepsilon \left(t\right)$ follows a skewed t-distribution with 5 degrees of freedom and the covariance function is $\gamma \left(s,t\right)=0.3\exp\left\{-\left|s-t\right|\right\}$, with a location parameter of ${\left(0,\ldots ,0\right)}^{T}$ and skewness parameter ${\left(2,\ldots ,2\right)}^{T}$.

#### 3.1.1. Example 1: Magnitude Outliers

The data generation process is defined as follows:

Normal sample: $X\left(t\right)=4t+U+\varepsilon \left(t\right)$, where U follows a uniform distribution $U\left(-1,\;1\right)$.

Anomalous sample: $X\left(t\right)=4t+T+\varepsilon \left(t\right)$, where T follows a uniform distribution $U\left(5,8\right)$ with a probability of 50% and $U\left(-8,-5\right)$ with a probability of 50%.

**Figure 4:**
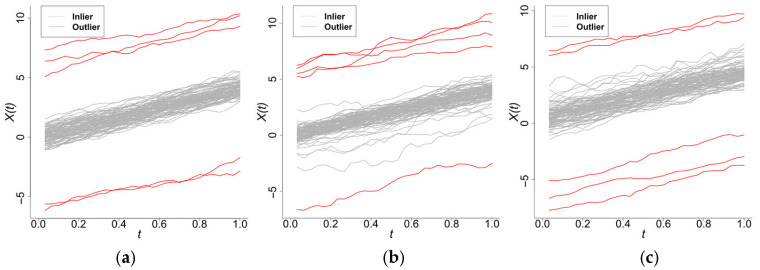
Data generated by Model 1, Model 2, and Model 3. (**a**) Model 1. (**b**) Model 2. (**c**) Model 3.

When $\varepsilon \left(t\right)$ follows scenarios 1, 2, or 3, they are referred to as Model 1, 2, or 3, respectively. The data (with an anomaly rate of 5%) are shown in Figure 4.

**Figure 5:**
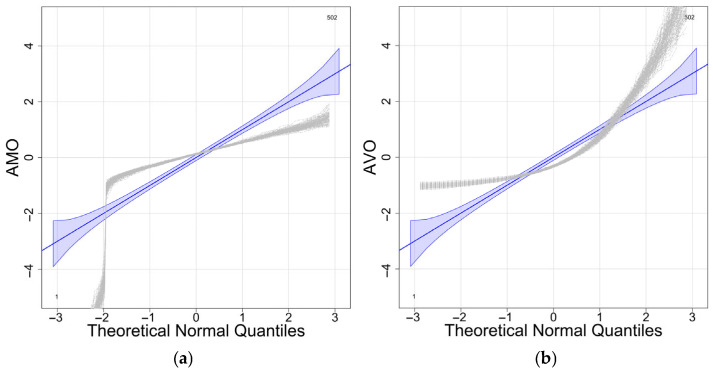
Normal Q-Q Plot for AMO and AVO (Model 3, c = 5%). (**a**) Q-Q Plot for AMO. (**b**) Q-Q Plot for AVO.

For W2, we assumed that the mean and variance of the outlyingness followed a multivariate normal distribution, with the Mahalanobis distance forming a statistic that follows an F-distribution. In Model 3 with multivariate skewed t-distributed errors, Figure 5 shows a clear violation of the multivariate normality assumption for the outlyingness distribution.

The proposed method demonstrated robust performance across all scenarios. It achieved a near-optimal balance between detection accuracy ($p_{c}$) and false positive control ($p_{f}$), as summarized in Table 1 and the tables in the Supplementary (standard deviations in parentheses). Notably, our approach consistently outperformed FAST-MUOD (W5) by maintaining a competitive $p_{c}$ while significantly reducing $p_{f}$ rates. Although the functional boxplot (W3) and bagplot (W4) methods achieved favorable $p_{c}$ and $p_{f}$ metrics, they lacked the adaptive capabilities of our technique in complex outlier configurations.

The proposed method demonstrated enhanced outlier detection capability under skewed *t*-distributions compared with standard *t*-distributions. Table 2 reports the average precision and F1 score of each method.

#### 3.1.2. Example 2: Persistent Shape Outliers

The data generation mechanism is as follows:

Normal sample: $X\left(t\right)=4t+\varepsilon \left(t\right)$.

Anomalous sample: $X\left(t\right)=4t+1.5\;sin\left(4\pi \left(t+\eta \right)\right)+\varepsilon \left(t\right),$ where η follows a uniform distribution $U\left(0.25,0.75\right)$.

When the error term $\varepsilon \left(t\right)$ follows scenarios 1, 2, or 3, the terms are referred to as Model 4, 5, or 6, respectively. The data sample plots (with an anomaly rate of 5%) are shown in Figure S1 in the Supplementary. Table 3 and Table S2 in the Supplementary summarize detection performance (standard deviations in parentheses) and demonstrate that our proposed method achieved consistently high accuracy comparable with W2 while exhibiting superior robustness across distributional scenarios. Notably, our proposed method maintained optimal performance under non-Gaussian conditions, whereas the competing methods deteriorated. Crucially, our method matched W2’s performance under Gaussian assumptions (Model 4), whereas it established clear superiority in non-Gaussian environments. Table 4 reports the average precision and F1 score of each method.

#### 3.1.3. Example 3: Isolated Shape Outliers

The data generation mechanism is as follows:

Normal sample:$\;X\left(t\right)=4t+\varepsilon \left(t\right)$.

Anomalous sample:$\;X\left(t\right)=4t+2U\times I_{T\leq t\leq T+0.05}+\varepsilon \left(t\right),$ where U has a 50% probability of being 1 and a 50% probability of being −1, and $I_{T\leq t\leq T+0.05}\;$ is an indicator function that takes the value 1 in the interval $\left[T,T+0.05\right]$ and 0 elsewhere. T follows a uniform distribution $U\left(0,0.8\right)$.

**Figure 6:**
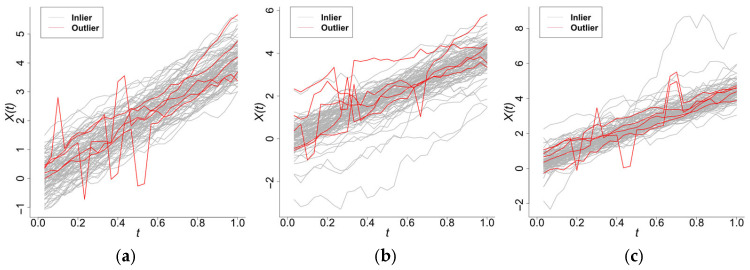
Sample plots of Model 0, Model 7, and Model 8. (**a**) Model 0. (**b**) Model 7. (**c**) Model 8.

When the error term $\varepsilon \left(t\right)$ follows scenario 1, the model is Model 0. When $\varepsilon \left(t\right)$ follows scenario 2 or 3, the models are referred to as Model 7 or 8, respectively. The sample plots (with an anomaly rate of 5%) are shown in Figure 6.

The detection results are presented in Table 5 and analogous patterns for sample size n=50 are shown in Supplementary Table S3. The proposed method demonstrated superior performance across all evaluation scenarios. It significantly outperformed W2–W5 in detection sensitivity while maintaining lower false positive rates. Crucially, our method exhibited consistently better performance than FAST-MUOD (W5) under all tested conditions. Moreover, the benchmark methods demonstrated significant degradation in $p_{c}$ values, which indicates their limited suitability for isolated shape outlier detection. Our proposed method has the best performance on precision and F1 score under this scenario, which is shown in Table 6. ROC curves of Model 8 are shown in the Supplementary File as an example.

#### 3.1.4. Example 4: Mixed Outliers

The data generation mechanism is as follows:

Normal sample:$\;X\left(t\right)=4t+\varepsilon \left(t\right)$.

Anomalous sample: (two types of anomalies mixed in equal proportions)

(1) $X\left(t\right)=4t+T+$ $\varepsilon \left(t\right)$, where T follows a uniform distribution $U\left(-5,-3\right)$.

(2) $X\left(t\right)=4t+1.5\sin\left(4\pi \left(t+\eta \right)\right)+\varepsilon \left(t\right)$, where η follows a uniform distribution $U\left(0.25,\;0.75\right)$.

When the error term $\varepsilon \left(t\right)$ follows scenarios 1, 2, or 3, the terms are referred to as Model 9, 10, or 11, respectively. The sample plots (with an anomaly rate of 5%, n=100) are shown in Figure S2 in the Supplementary.

As summarized in Table 7 (standard deviations in parentheses), our AO methods (W1_1 and W1_2) demonstrated robust performance across all scenarios. They achieved the optimal balance between high sensitivity and low false positive rates. Although competing methods demonstrated comparable sensitivity in most cases, critical deficiencies emerged in the false positive control: W2, W4, and W5 exhibited substantially elevated false positive rates, whereas W3’s conservative approach yielded unacceptable sensitivity reduction. This comparative analysis establishes our methodology’s distinct advantage in maintaining diagnostic precision without compromising detection power. Results with n=50 are shown in Table S4 in the Supplementary with a similar pattern. Precision and F1 score have similar patterns to the prevision examples. We put the record in the Supplementary File.

### 3.2. Multivariate Functional Data

There are two types for multivariate functional data outlier detection. The marginal approach applies detection methods dimension-wise to individual components $X_{i}^{j}\left(t\right)$,  j=1,…,p,i=1,…,n, thereby classifying curves as outliers when anomalies appear in any dimension. This constitutes a union operation over dimension-specific outliers. Conversely, the multivariate approach analyzes the complete functional structure using holistic multidimensional methodologies that preserve cross-dimensional dependencies. Our proposed method is implemented through both marginal (Mar.W1) and multivariate (Tot.W1) approaches with the bootstrap MCD robust Mahalanobis distance statistic. Similarly, the DO method [22] is executed marginally (Mar.W2) and multivariately (Tot.W2). Functional boxplot [34] and functional bagplot [24] methods lack inherent multivariate support and are thus applied only marginally (Mar.W3, Mar.W4). FAST-MUOD uses a random projection technique [19] (Prj.W5). This comparative structure enables equitable evaluation across methodological paradigms.

Models 12–15 were used in [35], which followed the setup of López–Pintado [36]. X(t) = e(t), where $e\left(t\right)=\{e_{1}\left(t\right),e_{2}{\left(t\right)\}}^{T}$ is a mean-zero bivariate Gaussian process with the cross-covariance function $C_{ij}\left(s,t\right)=\rho_{ij}\sigma_{i}\sigma_{j}M\left(\left|s-t\right|;\nu_{ij},\alpha_{ij}\right),\;i,j=1,2$, where $\rho_{12}$ represents the correlation between $X_{1}\left(t\right)$ and $X_{2}\left(t\right)$, $\rho_{11}=\rho_{22}=1,\sigma_{i}^{2}$ are the marginal variances, and $M\left(h;\nu ,\alpha \right)=2^{1-\nu }\Gamma {\left(\nu \right)}^{-1}{\left(\alpha \left|h\right|\right)}^{\nu }K_{\nu }\left(\alpha \left|h\right|\right)$ (with $\left|h\right|=\left|s-t\right|$) is the Matérn class [37], where $K_{\nu }$ is the modified Bessel function of the second kind. ν>0  is a smoothness parameter, and α>0 is a range parameter. Throughout the simulations, we used the following parameters for the bivariate Matérn cross-covariance function: $\sigma_{1}=\sigma_{2}=1,\;\alpha_{11}=0.02,\alpha_{22}=0.01,\alpha_{12}=0.016,\;\nu_{11}=1.2,\nu_{22}=0.6,\nu_{12}=1,\rho_{12}=0.6$.

Model 12 (Isolated outlier)

Normal sample: X(t) = e(t).

Anomalous sample: $X_{i}\left(t\right)=e_{i}\left(t\right)\left(1\pm 5I_{T\leq t\leq T+0.1}\right),\;i=1,\;2,$ where the sign is +5 if T>0.45 and −5 otherwise.

Model 13 (Shift outlier)

Baseline model: X(t) = e(t).

Contaminated model: $X_{i}\left(t\right)=e_{i}\left(t\right)+1.2,\;i=1,\;2$.

Model 14 (Consistent outlier)

Baseline model: X(t) = e(t).

Contaminated model: $X_{1}\left(t\right)=1.7e_{1}\left(t\right)$ and $X_{2}\left(t\right)=1.5e_{2}\left(t\right),i=1,2$.

Model 15 (Shape outlier)

Baseline model: $X_{1}\left(t\right)=e_{1}\left(t\right)+U_{11}\cos\left(4\pi t\right)$ and $X_{2}\left(t\right)=e_{2}\left(t\right)+U_{12}\sin\left(4\pi t\right)$, where $U_{11}$ and $U_{12}$ are independent and uniformly distributed on [2,3].

Contaminated model:$\;X_{1}\left(t\right)=e_{1}\left(t\right)+U_{21}\cos\left(4\pi t\right)$ and $X_{2}\left(t\right)=e_{2}\left(t\right)+U_{22}\sin\left(4\pi t\right)$, where $U_{21}$ and $U_{22}$ are independent and uniformly distributed on [4,5].

**Figure 7:**
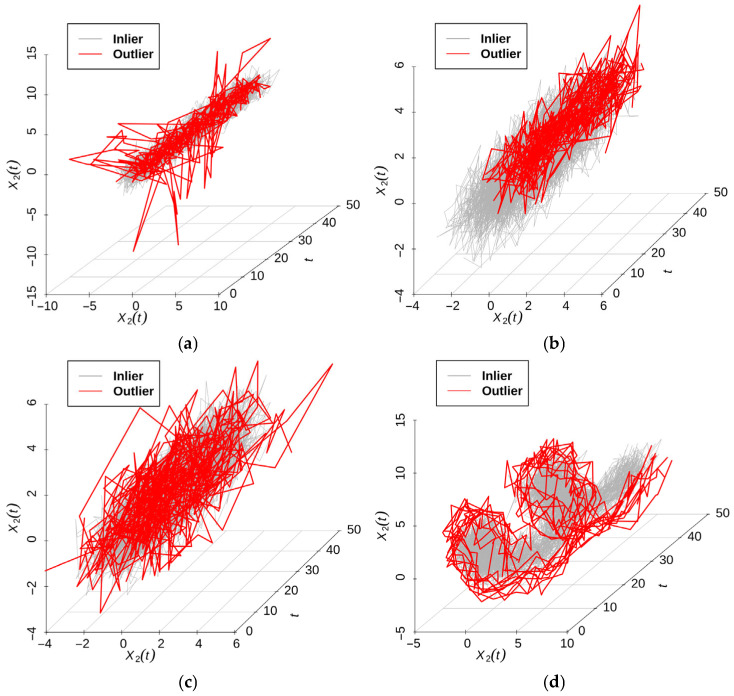
Samples of Model 12, Model 13, Model 14, and Model 15. (**a**) Model 12. (**b**) Model 13. (**c**) Model 14. (**d**) Model 15.

We set the contamination proportion in the contaminated model to c=0.1. The sample size was n=100, and we observed the samples at 50 equally spaced time points within the interval [0,1]. We replicated each simulation 100 times. Sample plots are shown in Figure 7. The results are summarized in Table 8.

Across Models 12–15, our proposed methods demonstrated superior performance. Mar.W1 achieved the highest sensitivity ($p_{c}$) for Models 12–14 and maintained competitive $p_{c}$ for Model 15. Tot.W1 consistently ranked second in $p_{c}\;$ for Models 12–14. Among the comparison methods, W2 yielded the lowest false positive rate ($p_{f}$), but exhibited reduced sensitivity. W3 failed to detect anomalies effectively across all models. Prj.W5 demonstrated moderate $p_{f}$ control, but suboptimal $p_{c}\;$ performance.

### 3.3. Simulation Results Summary

The proposed AOD method demonstrates significant advantages across diverse analytical scenarios. It maintains robust outlier detection capabilities under varying models, anomaly proportions, and sample sizes while effectively controlling false positive rates. Crucially, AOD performs reliably under non-Gaussian distributions where conventional methods deteriorate. For isolated shape outliers, AOD achieves significantly higher detection accuracy than benchmark methods. Our bootstrap minimum covariance determinant implementation demonstrates superior detection performance compared to the FAST-MCD variant, albeit with higher computational demands. Friedman test and Wilcoxon test are conducted on $p_{c}$ and $p_{f}$ superlatively on different outlier proportions with the methods compared. Our method has significantly better performance on $p_{c}$ and $p_{f}$. For details, please refer to the Supplementary File.

## 4. Application

### 4.1. Sea Water Spectral Data Outlier Detection

#### 4.1.1. Data Introduction

**Figure 8:**
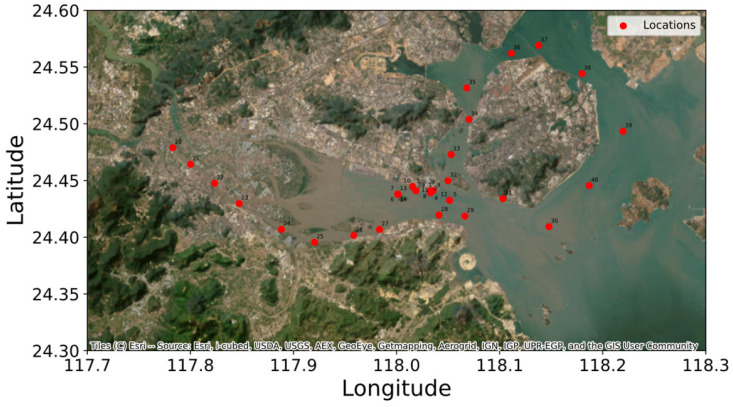
Seawater sampling locations. Red dots indicate sampling points, and we conducted the spectral characterization of seawater samples across a 190–1100 nm range at 1 nm resolution, with the resultant profiles presented in Figure 9. A qualitative assessment demonstrated four distinct spectral classes: representative baseline spectra (gray curves), magnitude-shifted profiles (blue curves), shape-variant spectra (red curves), and combined magnitude-shape anomalies (green curves). Consistent with established spectroscopic protocols for aqueous matrices, we restricted the analytical scope to the 190–600 nm spectral window. This selective spectral truncation mitigated interference from water absorption bands and detector noise amplification beyond 600 nm, thereby ensuring analytical fidelity during subsequent chemometric processing.

In an empirical analysis, we used seawater spectral data collected from coastal waters near Xiamen City, Fujian Province, China. The dataset comprised spectral measurements from 40 seawater samples obtained across three strategically selected regions: (1) the Jiulong River estuary, (2) the transitional zone from the estuary to the open sea, and (3) the surrounding waters of Xiamen Island. Figure 8 provides the precise geographical distribution of the sampling locations.

**Figure 9:**
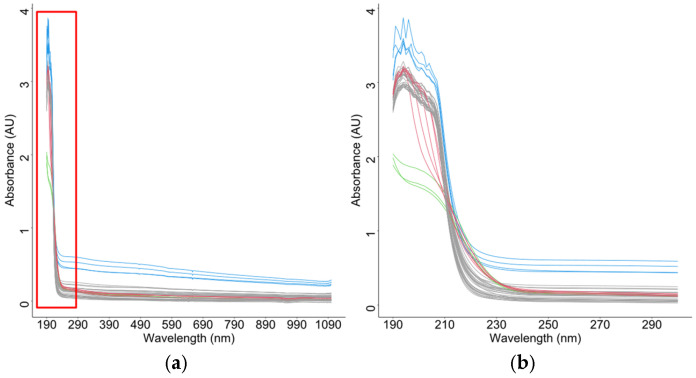
Seawater sample absorbance data. (**a**) Full spectrum from 190 nm to 1100 nm. (**b**) Zooming in on the wavelength range from 190 nm to 300 nm.

#### 4.1.2. Outlier Detection

**Figure 10:**
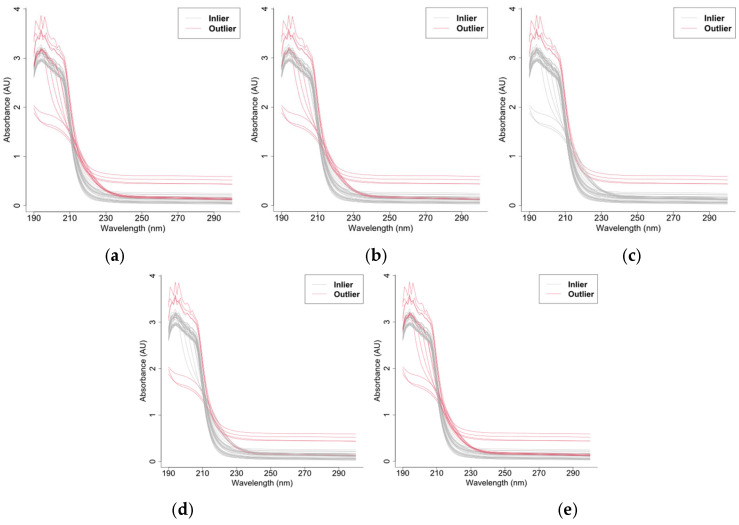
Seawater outlier detection results. (**a**) The adjusted outlyingness detection method. (**b**) The directional outlyingness detection method. (**c**) The functional boxplot. (**d**) The functional bagplot. (**e**) FAST-MUOD.

We performed outlier detection on seawater spectral data (40 samples) using the proposed AOD method (W1, W1_1, and W1_2 had the same result and are hence denoted by W1) and benchmark methods (W2–W5 as in the simulation). As shown in Figure 10, the proposed AOD method identified the most comprehensive set of outliers (samples 15–18 and 20–27). The comparative results demonstrated that DO (W2) detected samples 15–18 and 20–25; functional boxplot (W3) detected only samples 15–18; functional bagplot (W4) detected samples 15–18 and 20–22; and method W5 yielded identical results to AOD. This analysis demonstrates AOD’s superior sensitivity, particularly in detecting additional spectral anomalies (samples 26–27) missed by other approaches (W2, W3, and W4). Scatter plots of all method indices are provided in the Supplementary.

#### 4.1.3. Performance

To validate detection accuracy, we conducted a contextual analysis of sampling locations: Samples 15–18 originated near Xiamen’s S Power Plant, where potential industrial contamination may compromise spectral integrity. Samples 20–27 represented freshwater environments from the Jiulong River estuary, which are fundamentally distinct from marine spectral profiles. Therefore, we designated these 12 samples as validated outliers based on environmental discontinuity. Using this expert-verified ground truth, we calculated precision ($p_{c}$), false positive ($p_{f}$), F1 score, and AUC metrics for all detection methods. Comparative performance results are quantified in Table 9.

**Figure 11:**
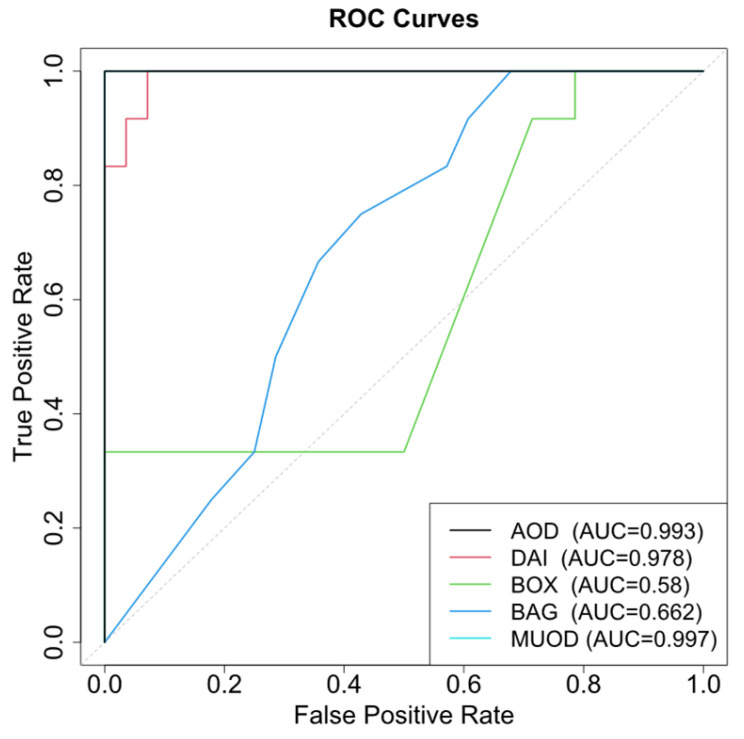
Outlier detection ROC curves.

As shown in Table 9, the proposed AOD method (W1) demonstrated superior performance in both sensitivity and false positive control compared with the benchmark methods. Its robustness in discriminating shape and positional outliers, coupled with comprehensive detection capabilities, established a particular efficacy for FDA. Although DO (W2) outperformed the functional boxplot (W3) and bagplot (W4) approaches, it exhibited detection deficiencies in complex outlier configurations. FAST-MUOD yielded identical results to AOD. ROC curves are in Figure 11.

Collectively, these results confirm that AOD and FAST-MUOD optimally enhanced inference accuracy through effective outlier removal.

### 4.2. Character Dataset Outlier Detection

**Figure 12:**
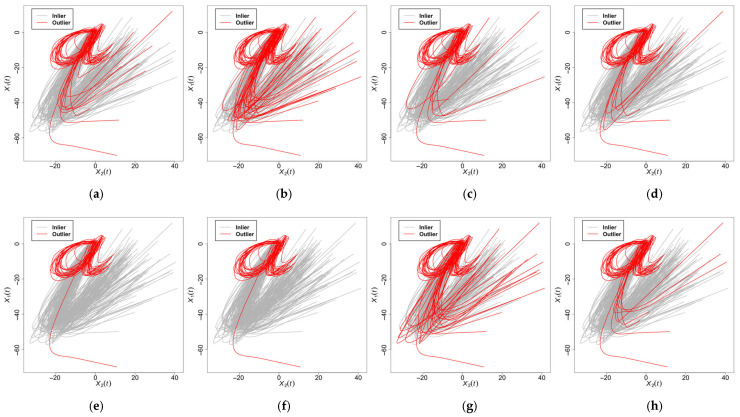
Character trajectory dataset data and results. (**a**) Character trajectory dataset data with outliers. (**b**) The adjusted outlyingness detection method marginally (**c**) The adjusted outlyingness detection method jointly. (**d**) The directional outlyingness detection method jointly. (**d**) The directional outlyingness detection method marginally. (**e**) The directional outlyingness detection method jointly. (**f**) The functional boxplot. (**g**) The functional bagplot. (**h**) FAST-MUOD.

We applied outlier detection to the character trajectory dataset analyzed by [19], originally sourced from the UCI Machine Learning Repository [38]. This multivariate dataset was comprised of 174 bivariate functional observations (pen-tip coordinates) recorded at 100 time points for the letter “i” Following established methodologies [19,39], 10 samples within this set were identified as consensus outliers. To augment the test environment, we incorporated 19 additional outlier samples from the letter “a” dataset (first 19 entries). The combined test set was comprised of 193 trajectories, including 29 confirmed anomalies (approximately 15%), thereby exhibiting structural heterogeneity relative to the base “i” distribution. Figure 12 illustrates both the complex trajectory patterns and detection outcomes.

We compared our results with the four benchmark methods, as conducted in the simulation, and obtained results which demonstrated our method’s capability to identify script anomalies across diverse handwriting profiles. Based on the detection results in Table 10, we drew the following conclusions regarding the proposed method’s performance on character trajectory data. The proposed AOD method marginally (Mar.W1) achieved the highest anomaly detection rate $p_{c}=89.7\%$, outperforming all benchmark methods. Although Tol.W2 and Mar.W3 achieved 0% false positives, their low sensitivity ($p_{c}\leq 68.9\%$) indicated high missed detection rates. Mar.W1 maintained a moderate false positive rate ($p_{f}=12.2\%$) comparable with Mar.W4. Additionally, FAST-MUOD (W5) exhibited suboptimal performance.

This analysis validates the method’s robustness for real-world handwriting analysis applications where writing style heterogeneity necessitates adaptable anomaly detection frameworks.

### 4.3. Outlier Detection for Population Data

**Figure 13:**
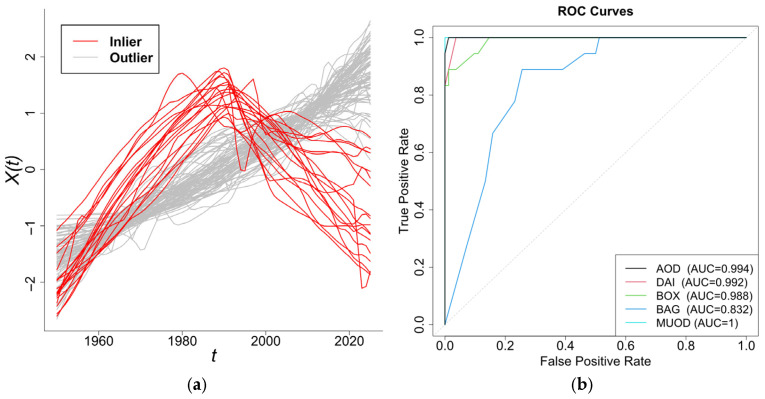
Outlier detection results on population data. (**a**) Normalized population curves with outliers. (**b**) ROC curves for population data outlier detection.

The data is sourced from the publicly available United Nations World Population Dataset” https://www.un.org/development/desa/pd/ (accessed on 11 November 2025)”. After filtering, it covers the total population estimates from 1950 to 2025 for 100 countries or regions across Europe and Asia, constituting 100 observation curves. The original data and its normalized results are shown in Figure 13. The data overall exhibits an upward trend. However, the population curves for some countries or regions show an initial increase, followed by a decline starting around 1990. We define these curves as outlier population curves; there are 18 such countries or regions (see Supplementary Materials for the list). Outlier detection was performed using the method proposed in this paper and several methods (W1–W5) mentioned in the simulation study. The results are presented in Table 11 and Figure 13.

The AOD method proposed in this paper and the FAST-MUOD method demonstrated superior performance. FAST-MUOD successfully identified all preset anomalous countries but also incorrectly flagged Qatar as an outlier. Based on its population curve, Qatar remains in an overall upward trend, which aligns more closely with the growth patterns of other normal samples and should not be classified as anomalous. In contrast, although the proposed AOD method missed one true anomaly, it resulted in zero false alarms. FAST-MUOD exhibited a higher false positive rate, which is consistent with findings from the earlier simulation study. F1 score shows the overall best performance by these two methods. Traditional methods such as boxplot and directional outlyingness approach missed several anomalies, indicating limited detection capability. The bagplot method not only performed poorly but also misclassified several clearly upward-trending countries as anomalies, reflecting its low sensitivity to variations in population curve shapes. In summary, the AOD method shows strong potential in achieving both high detection rates and low false positive rates.

## 5. Conclusions and Discussion

The detection and identification of anomalies in signals is a cornerstone of reliable information analysis. In functional data, this translates to the critical task of pinpointing aberrant curves that deviate from the dominant process, a process essential for data integrity. Accurately achieving this using probabilistic, statistical, and information-theoretic principles is paramount, as it allows us to formally quantify the “unexpectedness” or information gain associated with anomalous patterns, moving beyond purely geometric distance metrics. In this study, we proposed the AOD method, which transforms the functional data outlier detection problem into a multivariate paradigm. The core innovation lies in the AO representation—comprising the Adjusted Mean, Variance, and Skewness of Directional Outlyingness $\left(AMO,AVO,SO\right)$—which achieves significant dimensionality reduction while preserving critical functional features. This representation, combined with bootstrap-based distribution estimation and the robust Mahalanobis distance, forms an integrated framework that overcomes the limitations of restrictive data distribution assumptions.

Our approach provided a detailed characterization of the magnitude and shape information of functional data. Metrologically, our method enhances measurement accuracy by assuring functional data quality through robust outlier detection, while the bootstrap-based threshold offers a data-driven uncertainty evaluation for the diagnostic process itself, establishing it as a reliable tool for validating measurements under non-ideal non-Gaussian conditions.

Simulation studies demonstrated that AOD performs best in detecting isolated and mixed shape outliers. It also alleviated the distributional assumption, which enhances its practical applicability—a claim verified by an empirical analysis of seawater spectral data. A limitation was observed at extreme outlier proportions (≥10%), where performance degraded in line with statistical intuition and benchmark methods. For such high-contamination scenarios, we recommend using a lower quantile threshold (e.g., 0.95 instead of 0.975) to maintain detection precision. This aligned with statistical intuition and was consistent with all benchmarks.

In practice, many data collection processes naturally yield observations at regular time points (e.g., spectral data), or the data can be obtained in a regular format through alignment. The AOD method can be directly applied to analyze such data. However, the AOD method cannot be directly applied to functional data with different sampling sequences (irregular grids) in its raw form. Unlike [40], which models the continuous latent process to handle irregularity, the AOD method implicitly requires a pre-processing step to align all curves onto a common grid using smoothing or interpolation. What is more, in the context of this paper, ‘Non-Gaussian’ refers to asymmetry (skewness) and heavy tails, not multi-modality. Consequently, the proposed metric does not accommodate multi-modal scenarios. If the dataset contains multiple valid modes (e.g., two different ‘normal’ operating conditions), the AOD method, which aggregates data into a single adjusted outlyingness vector and calculates a distance from a global center, will likely misclassify samples from the smaller (or further) mode as outliers. To handle such scenarios, a mixture model structure or a pre-processing step involving functional clustering would be required before applying AOD. These are valuable directions that warrant further research in our future work.

Future research directions include incorporating higher-order derivatives and moments to enhance anomaly detection capabilities: developing more efficient resampling strategies, such as subsampling or parallelized bootstrap algorithms, to improve computational efficiency without sacrificing statistical accuracy. Applying the complete clustering-detection-classification framework [9] to the AO metric vector and exploring methods for scenarios where normal data exhibit subgroup structures is another potential extension. While recent literature has moved toward explicit classification of anomaly types, real-world functional data often exhibits complex or hybrid patterns that defy pre-defined categories [19]. Consequently, our approach aligns with generalized frameworks, like those of Al Samara et al. [9] and Dai and Genton [22], by prioritizing robust, universal detection over rigid categorization. Future work will explore how components of the AOD vector could be adapted to provide more granular diagnostic insights without sacrificing this flexibility.

Furthermore, we can consider the proposed method for batch processing scenarios, such that future research could address its scalability to very large datasets and its extension to an online, real-time detection framework. The detection threshold is established using a representative subset of the data, it can be applied consistently to new or larger datasets without repeating the bootstrap resampling. This makes the anomaly detection for a large dataset computationally efficient. Although not covered in this study, other anomaly detection methods, such as time series data, present theoretical ideas of considerable merit.

## Figures and Tables

**Table 1:** Example 1 result (standard deviations in parentheses, *n* = 100).

		c = 1%		c = 5%		c = 10%	
		$p_{c}$	$p_{f}$	$p_{c}$	$p_{f}$	$p_{c}$	$p_{f}$
Model 1	W1_1	100.0%	3.5%	100.0%	2.9%	100.0%	2.1%
		(0.0000)	(0.0236)	(0.0000)	(0.0217)	(0.0000)	(0.0178)
	W1_2	100.0%	2.2%	100.0%	1.9%	99.2%	1.5%
		(0.0000)	(0.0242)	(0.0000)	(0.0221)	(0.0393)	(0.0198)
	W2	100.0%	2.7%	100.0%	2.5%	100.0%	2.2%
		(0.0000)	(0.0231)	(0.0000)	(0.0218)	(0.0000)	(0.0207)
	W3	100.0%	0.02%	100.0%	0.01%	100.0%	0.01%
		(0.0000)	(0.0014)	(0.0000)	(0.0010)	(0.0000)	(0.0011)
	W4	100.0%	7.7%	100.0%	6.8%	100.0%	5.7%
		(0.0000)	(0.0369)	(0.0000)	(0.0297)	(0.0000)	(0.0284)
	W5	100.0%	8.0%	100.0%	7.5%	100.0%	7.2%
		(0.0000)	(0.0253)	(0.0000)	(0.0259)	(0.0000)	(0.0275)
Model 2	W1_1	100.0%	7.9%	100.0%	6.9%	99.4%	5.7%
		(0.0000)	(0.0307)	(0.0000)	(0.0293)	(0.0277)	(0.0272)
	W1_2	100.0%	8.3%	99.8%	7.3%	99.0%	6.0%
		(0.0000)	(0.0322)	(0.0200)	(0.0295)	(0.0481)	(0.0279)
	W2	100.0%	7.8%	100.0%	7.4%	99.9%	6.7%
		(0.0000)	(0.0336)	(0.0000)	(0.0316)	(0.0100)	(0.0328)
	W3	100.0%	1.8%	100.0%	1.7%	100.0%	1.4%
		(0.0000)	(0.0149)	(0.0000)	(0.0150)	(0.0342)	(0.0133)
	W4	100.0%	13.8%	100.0%	12.6%	100.0%	11.2%
		(0.0000)	(0.0365)	(0.0000)	(0.0325)	(0.0000)	(0.0345)
	W5	100.0%	12.5%	100.0%	12.1%	100.0%	11.6%
		(0.0000)	(0.0295)	(0.0000)	(0.0288)	(0.0000)	(0.0324)
Model 3	W1_1	100.0%	8.5%	97.6%	7.4%	97.0%	5.8%
		(0.0000)	(0.0337)	(0.0883)	(0.0323)	(0.0732)	(0.0286)
	W1_2	100.0%	9.0%	98.2%	8.0%	94.4%	6.5%
		(0.0000)	(0.0357)	(0.0757)	(0.0357)	(0.1472)	(0.0317)
	W2	100.0%	8.9%	99.8%	8.2%	99.6%	7.5%
		(0.0000)	(0.0305)	(0.0200)	(0.0275)	(0.0197)	(0.0323)
	W3	100.0%	0.7%	100.0%	0.6%	100.0%	0.4%
		(0.0000)	(0.0087)	(0.0000)	(0.0086)	(0.0000)	(0.0074)
	W4	100.0%	8.7%	100.0%	8.3%	100.0%	7.7%
		(0.0000)	(0.0304)	(0.0000)	(0.0271)	(0.0000)	(0.0302)
	W5	100.0%	11.9%	100.0%	11.4%	100.0%	11.5%
		(0.0000)	(0.0264)	(0.0000)	(0.0271)	(0.0000)	(0.0305)

**Table 2:** Example 1 result on precision and F1 score.

		c=1%		c=5%		c=10%	
		Precision	$F_{1}$	Precision	$F_{1}$	Precision	$F_{1}$
Model 1	W1_1	0.22	0.37	0.64	0.78	0.84	0.91
	W1_2	0.31	0.48	0.73	0.85	0.88	0.93
	W2	0.27	0.43	0.68	0.81	0.83	0.91
	W3	0.98	0.99	1.00	1.00	1.00	1.00
	W4	0.12	0.21	0.44	0.61	0.66	0.80
	W5	0.11	0.20	0.41	0.58	0.61	0.76
Model 2	W1_1	0.11	0.2	0.43	0.60	0.66	0.79
	W1_2	0.11	0.2	0.42	0.59	0.65	0.78
	W2	0.11	0.21	0.42	0.59	0.62	0.77
	W3	0.36	0.53	0.76	0.86	0.89	0.94
	W4	0.07	0.13	0.29	0.46	0.50	0.66
	W5	0.07	0.14	0.3	0.47	0.49	0.66
Model 3	W1_1	0.11	0.19	0.41	0.58	0.65	0.78
	W1_2	0.10	0.18	0.39	0.56	0.62	0.75
	W2	0.10	0.18	0.39	0.56	0.60	0.75
	W3	0.59	0.74	0.90	0.95	0.97	0.98
	W4	0.10	0.19	0.39	0.56	0.59	0.74
	W5	0.08	0.15	0.32	0.48	0.49	0.66

**Table 3:** Example 2 result (standard deviations in parentheses, n=100).

		c = 1%		c = 5%		c = 10%	
		$p_{c}$	$p_{f}$	$p_{c}$	$p_{f}$	$p_{c}$	$p_{f}$
Model 4	W1_1	100.0%	3.7%	100.0%	3.1%	100.0%	2.1%
		(0.0000)	(0.0213)	(0.0000)	(0.0200)	(0.0000)	(0.0170)
	W1_2	100.0%	3.8%	100.0%	3.2%	100.0%	2.4%
		(0.0000)	(0.0223)	(0.0000)	(0.0208)	(0.0000)	(0.0181)
	W2	100.0%	2.6%	100.0%	2.6%	100.0%	2.2%
		(0.0000)	(0.0205)	(0.0000)	(0.0229)	(0.0000)	(0.0221)
	W3	16.0%	0.3%	13.8%	0.2%	10.7%	0.1%
		(0.3685)	(0.0017)	(0.1650)	(0.0014)	(0.1281)	(0.0011)
	W4	18.0%	8.0%	26.2%	7.7%	30.5%	6.8%
		(0.3861)	(0.0371)	(0.2390)	(0.0358)	(0.1888)	(0.0307)
	W5	100.0%	7.8%	100.0%	6.3%	100.0%	4.6%
		(0.0000)	(0.0253)	(0.0000)	(0.0248)	(0.0000)	(0.0221)
Model 5	W1_1	100.0%	7.8%	100.0%	7.0%	100.0%	5.8%
		(0.0000)	(0.0303)	(0.0000)	(0.0282)	(0.0000)	(0.0282)
	W1_2	100.0%	8.2%	100.0%	7.2%	100.0%	6.0%
		(0.0000)	(0.0311)	(0.0000)	(0.0292)	(0.0000)	(0.0279)
	W2	100.0%	7.8%	100.0%	7.4%	100.0%	6.6%
		(0.0000)	(0.0337)	(0.0000)	(0.0327)	(0.0000)	(0.0325)
	W3	16.0%	2.0%	15.4%	1.7%	11.5%	1.5%
		(0.3685)	(0.0154)	(0.1749)	(0.0156)	(0.1344)	(0.0135)
	W4	27.0%	14.3%	27.2%	13.4%	30.5%	12.4%
		(0.4461)	(0.0346)	(0.2336)	(0.0342)	(0.1635)	(0.0356)
	W5	100.0%	12.1%	100.0%	10.6%	99.9%	8.8%
		(0.0000)	(0.0288)	(0.0000)	(0.0274)	(0.0100)	(0.0271)
Model 6	W1_1	100.0%	9.9%	100.0%	8.9%	100.0%	7.4%
		(0.0000)	(0.0249)	(0.0000)	(0.0269)	(0.0000)	(0.0273)
	W1_2	100.0%	10.3%	100.0%	8.9%	100.0%	7.8%
		(0.0000)	(0.0267)	(0.0000)	(0.0260)	(0.0000)	(0.0268)
	W2	100.0%	8.2%	100.0%	7.8%	100.0%	7.2%
		(0.0000)	(0.0289)	(0.0000)	(0.0290)	(0.0000)	(0.0303)
	W3	23.0%	2.1%	20.4%	1.8%	18.5%	1.8%
		(0.4229)	(0.0141)	(0.1681)	(0.0134)	(0.1395)	(0.0128)
	W4	22.0%	10.6%	29.6%	9.8%	31.5%	9.0%
		(0.4163)	(0.0284)	(0.2117)	(0.0276)	(0.1725)	(0.0298)
	W5	100.0%	12.2%	100.0%	10.5%	99.9%	8.7%
		(0.0000)	(0.0270)	(0.0000)	(0.0244)	(0.0100)	(0.0221)

**Table 4:** Example 2 results on precision and F1 score.

		c=1%		c=5%		c=10%	
		Precision	$F_{1}$	Precision	$F_{1}$	Precision	$F_{1}$
Model 4	W1_1	0.21	0.35	0.63	0.77	0.84	0.91
	W1_2	0.21	0.35	0.62	0.77	0.82	0.90
	W2	0.28	0.44	0.67	0.80	0.83	0.91
	W3	0.35	0.22	0.78	0.23	0.92	0.19
	W4	0.02	0.04	0.15	0.19	0.33	0.32
	W5	0.11	0.21	0.46	0.63	0.71	0.83
Model 5	W1_1	0.11	0.21	0.43	0.60	0.66	0.79
	W1_2	0.11	0.20	0.42	0.59	0.65	0.79
	W2	0.11	0.21	0.42	0.59	0.63	0.77
	W3	0.07	0.10	0.32	0.21	0.46	0.18
	W4	0.02	0.04	0.10	0.14	0.21	0.25
	W5	0.08	0.14	0.33	0.50	0.56	0.72
Model 6	W1_1	0.09	0.17	0.37	0.54	0.60	0.75
	W1_2	0.09	0.16	0.37	0.54	0.59	0.74
	W2	0.11	0.20	0.40	0.57	0.61	0.76
	W3	0.10	0.14	0.37	0.26	0.53	0.27
	W4	0.02	0.04	0.14	0.19	0.28	0.30
	W5	0.08	0.14	0.33	0.50	0.56	0.72

**Table 5:** Example 3 result (standard deviations in parentheses, n=100).

			c = 1%	c = 5%		c = 10%	
		$p_{c}$	$p_{f}$	$p_{c}$	$p_{f}$	$p_{c}$	$p_{f}$
Model 0	W1_1	93.0%	3.8%	95.6%	3.3%	95.4%	3.0%
		(0.2564)	(0.0228)	(0.0924)	(0.0213)	(0.0771)	(0.0204)
	W1_2	99.0%	4.0%	95.8%	3.7%	92.7%	3.0%
		(0.1000)	(0.0227)	(0.0818)	(0.0221)	(0.0930)	(0.0201)
	W2	51.0%	2.8%	41.2%	2.6%	38.1%	2.1%
		(0.5024)	(0.0206)	(0.2571)	(0.0216)	(0.2049)	(0.0191)
	W3	19.0%	0.04%	12.2%	0.04%	11.6%	0.3%
		(0.3943)	(0.0019)	(0.1447)	(0.0020)	(0.1032)	(0.0019)
	W4	13.0%	8.0%	9.2%	8.1%	10.4%	7.6%
		(0.3379)	(0.0366)	(0.1220)	(0.0349)	(0.0963)	(0.0350)
	W5	91.0%	8.0%	87.2%	6.6%	80.8%	5.2%
		(0.2880)	(0.0265)	(0.1650)	(0.0258)	(0.1570)	(0.0240)
Model 7	W1_1	78.0%	8.0%	74.6%	7.4%	68.9%	6.7%
		(0.4163)	(0.0308)	(0.2664)	(0.0294)	(0.2242)	(0.0315)
	W1_2	80.0%	8.3%	76.8%	7.8%	69.7%	7.0%
		(0.4020)	(0.0322)	(0.2473)	(0.0309)	(0.2119)	(0.0312)
	W2	38.0%	7.8%	29.4%	7.3%	24.6%	6.2%
		(0.4878)	(0.0330)	(0.2486)	(0.0328)	(0.1919)	(0.0317)
	W3	7.0%	2.0%	11.0%	1.9%	10.1%	1.9%
		(0.2564)	(0.0157)	(0.1432)	(0.0151)	(0.0969)	(0.0158)
	W4	14.0%	13.9%	16.6%	14.1%	15.5%	13.8%
		(0.3488)	(0.0357)	(0.1558)	(0.0409)	(0.1086)	(0.0353)
	W5	69.0%	12.3%	58.8%	11.3%	46.5%	9.9%
		(0.4650)	(0.0287)	(0.2480)	(0.0281)	(0.1940)	(0.0302)
Model 8	W1_1	96.0%	10.1%	90.8%	9.7%	88.2%	8.9%
		(0.1969)	(0.0252)	(0.1346)	(0.0301)	(0.1252)	(0.0294)
	W1_2	96.0%	10.6%	90.0%	9.9%	88.5%	9.3%
		(0.1969)	(0.0262)	(0.1435)	(0.0316)	(0.1236)	(0.0295)
	W2	43.0%	8.3%	36.2%	7.7%	28.5%	6.7%
		(0.4975)	(0.0283)	(0.2407)	(0.0300)	(0.1851)	(0.0314)
	W3	21.0%	2.1%	18.6%	2.0%	16.1%	2.0%
		(0.4093)	(0.0141)	(0.1712)	(0.0139)	(0.1311)	(0.0126)
	W4	24.0%	10.5%	15.4%	10.6%	15.5%	10.6%
		(0.4292)	(0.0264)	(0.1500)	(0.0265)	(0.1081)	(0.0309)
	W5	65.0%	12.3%	61.2%	10.9%	48.9%	9.5%
		(0.4790)	(0.0267)	(0.2480)	(0.0238)	(0.1850)	(0.0235)

**Table 6:** Example 3 result on precision and F1 score.

		c=1%		c=5%		c=10%	
		Precision	$F_{1}$	Precision	$F_{1}$	Precision	$F_{1}$
Model 0	W1_1	0.20	0.33	0.60	0.74	0.78	0.86
	W1_2	0.20	0.33	0.58	0.72	0.77	0.84
	W2	0.16	0.24	0.45	0.43	0.67	0.49
	W3	0.83	0.31	0.94	0.22	0.81	0.20
	W4	0.02	0.03	0.06	0.07	0.13	0.12
	W5	0.10	0.19	0.41	0.56	0.63	0.71
Model 7	W1_1	0.09	0.16	0.35	0.47	0.53	0.60
	W1_2	0.09	0.16	0.34	0.47	0.53	0.60
	W2	0.05	0.08	0.17	0.22	0.31	0.27
	W3	0.03	0.05	0.23	0.15	0.37	0.16
	W4	0.01	0.02	0.06	0.09	0.11	0.13
	W5	0.05	0.10	0.21	0.31	0.34	0.39
Model 8	W1_1	0.09	0.16	0.33	0.48	0.52	0.66
	W1_2	0.08	0.15	0.32	0.48	0.51	0.65
	W2	0.05	0.09	0.20	0.26	0.32	0.30
	W3	0.09	0.13	0.33	0.24	0.47	0.24
	W4	0.02	0.04	0.07	0.10	0.14	0.15
	W5	0.05	0.09	0.23	0.33	0.36	0.42

**Table 7:** Mixed outlier detection result (standard deviations in parentheses, n=100).

			c = 1%	c = 5%		c = 10%	
		$p_{c}$	$p_{f}$	$p_{c}$	$p_{f}$	$p_{c}$	$p_{f}$
Model 9	W1_1	99.0%	3.4%	77.8%	2.8%	94.1%	1.9%
		(0.1000)	(0.0232)	(0.0746)	(0.0212)	(0.0922)	(0.0165)
	W1_2	92.0%	3.7%	76.6%	3.1%	93.4%	2.0%
		(0.2726)	(0.0241)	(0.0806)	(0.0236)	(0.1046)	(0.0167)
	W2	87.0%	2.7%	76.6%	2.6%	94.5%	2.0%
		(0.3379)	(0.0235)	(0.0901)	(0.0220)	(0.0687)	(0.0190)
	W3	100.0%	0.2%	44.8%	0.2%	54.1%	0.1%
		(0.0000)	(0.0014)	(0.0904)	(0.0014)	(0.0683)	(0.0011)
	W4	100.0%	7.7%	50.2%	7.4%	63.4%	6.4%
		(0.0000)	(0.0335)	(0.1317)	(0.0327)	(0.1216)	(0.0316)
	W5	100.0%	7.9%	80.4%	7.2%	100.0%	5.6%
		(0.0000)	(0.0266)	(0.0281)	(0.0291)	(0.0000)	(0.0245)
Model 10	W1_1	100.0%	7.1%	63.8%	5.8%	66.5%	4.3%
		(0.0000)	(0.0271)	(0.1674)	(0.0231)	(0.1641)	(0.0224)
	W1_2	86.1%	8.2%	75.6%	7.3%	86.3%	5.7%
		(0.3472)	(0.0320)	(0.1380)	(0.0308)	(0.1580)	(0.0269)
	W2	92.0%	7.8%	78.2%	7.4%	92.4%	6.7%
		(0.2714)	(0.0335)	(0.1140)	(0.0307)	(0.1045)	(0.0325)
	W3	99.0%	1.9%	46.8%	1.8%	56.0%	1.4%
		(0.0995)	(0.0154)	(0.1155)	(0.0151)	(0.0777)	(0.0141)
	W4	100.0%	13.9%	54.8%	13.5%	65.6%	12.1%
		(0.0000)	(0.0350)	(0.1573)	(0.0346)	(0.1139)	(0.0320)
	W5	100.0%	12.6%	82.8%	11.6%	99.8%	9.8%
		(0.0000)	(0.0292)	(0.0697)	(0.0280)	(0.0141)	(0.0280)
Model 11	W1_1	100.0%	10.0%	81.2%	9.4%	100.0%	7.9%
		(0.0000)	(0.0317)	(0.04773)	(0.0312)	(0.0000)	(0.0283)
	W1_2	100.0%	10.4%	81.2%	9.7%	100.0%	8.1%
		(0.0000)	(0.0317)	(0.04773)	(0.0324)	(0.0000)	(0.0291)
	W2	99.0%	8.5%	80.8%	8.0%	100.0%	7.4%
		(0.1000)	(0.0333)	(0.0393)	(0.0324)	(0.0000)	(0.0319)
	W3	99.0%	2.0%	49.4%	1.9%	60.2%	2.0%
		(0.1000)	(0.0157)	(0.1153)	(0.0150)	(0.0840)	(0.0158)
	W4	100.0%	10.5%	49.6%	10.5%	61.5%	10.8%
		(0.0000)	(0.0322)	(0.1377)	(0.0313)	(0.0892)	(0.0325)
	W5	99.0%	12.5%	82.0%	11.7%	100.0%	10.3%
		(0.1000)	(0.0263)	(0.0603)	(0.0258)	(0.0000)	(0.0255)

**Table 8:** Results on Models 12–15 (standard deviations in parentheses, n=100).

	Model 12		Model 13		Model 14		Model 15	
	$p_{c}$	$p_{f}$	$p_{c}$	$p_{f}$	$p_{c}$	$p_{f}$	$p_{c}$	$p_{f}$
Mar.W1	99.7%	10.1%	99.9%	4.6%	99.1%	5.8%	98.8%	5.6%
	(0.0171)	(0.0316)	(0.0100)	(0.0231)	(0.0321)	(0.0271)	(0.0327)	(0.02935)
Tot.W1	98.8%	1.1%	99.0%	0.3%	96.6%	0.4%	96.0%	0.3%
	(0.0356)	(0.0117)	(0.0333)	(0.0066)	(0.0476)	(0.0074)	(0.0550)	(0.0062)
Mar.W2	82.9%	0.0%	56.5%	0.07%	61.1%	0.01%	67.0%	0.0%
	(0.1297)	(0.0000)	(0.2330)	(0.0028)	(0.1870)	(0.0011)	(0.1700)	(0.0000)
Tot.W2	93.8%	0.02%	69.8%	0.0%	84.5%	0.01%	83.4%	0.02%
	(0.0873)	(0.0015)	(0.2010)	(0.0000)	(0.1180)	(0.0011)	(0.1480)	(0.0015)
Mar.W3	49.5%	0.0%	0.0%	0.0%	0.5%	0.0%	0.0%	0.0%
	(0.1560)	(0.0000)	(0.0000)	(0.0000)	(0.0219)	(0.0000)	(0.0000)	(0.0000)
Mar.W4	54.3%	7.6%	97.1%	7.6%	49.4%	7.1%	98.0%	7.4%
	(0.1790)	(0.0210)	(0.0795)	(0.0269)	(0.1699)	(0.0222)	(0.0620)	(0.0290)
Prj.W5	38.7%	2.6%	98.2%	1.7%	29.3%	2.6%	99.8%	3.9%
	(0.1610)	(0.0158)	(0.0435)	(0.0134)	(0.1370)	(0.0179)	(0.0141)	(0.0208)

**Table 9:** Detection results for seawater spectral data.

Method	Anomalous Samples	$p_{c}$	$p_{f}$	Precision	$F_{1}$	AUC
W1	15, 16, 17, 18, 20, 21, 22, 23, 24, 25, 26, 27	100.0%	0.00%	1.000	1.000	0.993
W2	15, 16, 17, 18, 20, 21, 22, 23, 24, 25	83.3%	0.00%	1.000	0.909	0.940
W3	15, 16, 17, 18	33.3%	0.00%	1.000	0.500	0.570
W4	15, 16, 17, 18, 20, 21, 22	58.3%	0.00%	1.000	0.737	0.662
W5	15, 16, 17, 18, 20, 21, 22, 23, 24, 25, 26, 27	100.0%	0.00%	1.000	1.000	0.997

**Table 10:** Detection results for character trajectory dataset data.

	Method	Mar.W1	Tol.W1	Mar.W2	Tol.W2	Mar.W3	Mar.W4	Prj.W5
	
$p_{c}$		89.70%	79.30%	82.70%	68.90%	68.90%	82.70%	79.30%
$p_{f}$		12.20%	1.80%	1.20%	0%	0%	12.20%	2.40%

**Table 11:** Outlier detection results.

Method	TPR ( $P_{c}$)	FPR ( $p_{f}$)	Precision	$F_{1}$	AUC
W1	0.9444	0.0000	1.0000	0.9714	0.994
W2	0.7778	0.0000	1.0000	0.8750	0.992
W3	0.6667	0.0000	1.0000	0.8000	0.988
W4	0.2778	0.0488	0.5556	0.3704	0.832
W5	1.0000	0.01220	0.9473	0.9730	0.999

## Data Availability

Data will be made available on request.

## Supplementary Materials

The Supplementary Materials can be downloaded at: https://www.mdpi.com/article/10.3390/e28020233/s1.
