# Supplementary material for: Outlier Detection in Functional Data Using Adjusted Outlyingness
Source: Entropy (Basel). 2026 Feb 16;28(2):233. doi: 10.3390/e28020233 (PMC12939932; doi:10.3390/e28020233)
Supplement: Supplementary file 1 [file entropy-28-00233-s001.zip › entropy-4097319-supplementary.pdf]

Supplementary of "Outlier Detection in Functional Data using Adjusted Outlyingness"

1. Simulation results with figures and tables

Table S1. Example 1 result (Standard Deviations in Parentheses, $n = 50$)

		c=2%		c=4%		c=10%	
		p_c	p_f	p_c	p_f	p_c	p_f
Model 1	W1_1	100.0%	1.8%	100.0%	1.7%	100.0%	1.4%
		(0.0000)	(0.0219)	(0.0000)	(0.0214)	(0.0000)	(0.0195)
	W1_2	100.0%	2.2%	100.0%	1.9%	99.2%	1.5%
		(0.0000)	(0.0242)	(0.0000)	(0.0221)	(0.0393)	(0.0198)
	W2	100.0%	2.4%	100.0%	2.4%	100.0%	2.1%
		(0.0000)	(0.0271)	(0.0000)	(0.0265)	(0.0000)	(0.0239)
	W3	100.0%	0.3%	100.0%	0.4%	100.0%	0.2%
		(0.0000)	(0.0094)	(0.0000)	(0.0096)	(0.0000)	(0.0063)
	W4	100.0%	9.0%	100.0%	8.7%	100.0%	7.3%
		(0.0000)	(0.0470)	(0.0000)	(0.0438)	(0.0000)	(0.0443)
	W5	100.0%	8.2%	100.0%	7.6%	100.0%	6.9%
		(0.0000)	(0.0375)	(0.0000)	(0.0335)	(0.0000)	(0.0368)
Model 2	W1_1	100.0%	5.4%	98.5%	5.0%	98.2%	4.5%
		(0.0000)	(0.0338)	(0.0857)	(0.0332)	(0.0808)	(0.0350)
	W1_2	100.0%	5.8%	98.0%	5.7%	98.2%	4.8%
		(0.0000)	(0.0352)	(0.0984)	(0.0361)	(0.0757)	(0.0377)
	W2	100.0%	5.8%	100.0%	6.5%	100.0%	6.0%
		(0.0000)	(0.0352)	(0.0000)	(0.0423)	(0.0000)	(0.0449)
	W3	100.0%	4.1%	100.0%	3.9%	100.0%	3.4%
		(0.0000)	(0.0363)	(0.0000)	(0.0354)	(0.0000)	(0.0341)
	W4	100.0%	14.3%	100.0%	13.6%	100.0%	12.4%
		(0.0000)	(0.0563)	(0.0000)	(0.0572)	(0.0000)	(0.0572)
	W5	100.0%	12.5%	100.0%	12.3%	100.0%	11.2%
		(0.0000)	(0.0407)	(0.0000)	(0.0404)	(0.0000)	(0.0443)
Model 3	W1_1	95.0%	4.4%	95.0%	4.2%	93.2%	3.4%
		(0.2190)	(0.0364)	(0.1946)	(0.0337)	(0.1890)	(0.0277)
	W1_2	95.0%	5.0%	94.0%	4.8%	90.6%	3.7%
		(0.2190)	(0.0374)	(0.2044)	(0.0355)	(0.2058)	(0.0299)
	W2	99.0%	6.8%	99.0%	6.3%	98.6%	5.6%
		(0.1000)	(0.0471)	(0.0703)	(0.0442)	(0.0651)	(0.0383)
	W3	100.0%	1.2%	100.0%	1.2%	100.0%	1.2%
		(0.0000)	(0.0164)	(0.0000)	(0.0169)	(0.0000)	(0.0171)
	W4	100.0%	9.6%	100.0%	9.7%	100.0%	8.9%
		(0.0000)	(0.0427)	(0.0000)	(0.0474)	(0.0000)	(0.0403)
	W5	100.0%	11.7%	100.0%	11.5%	100.0%	11.1%
		(0.0000)	(0.0403)	(0.0000)	(0.0425)	(0.0000)	(0.0470)

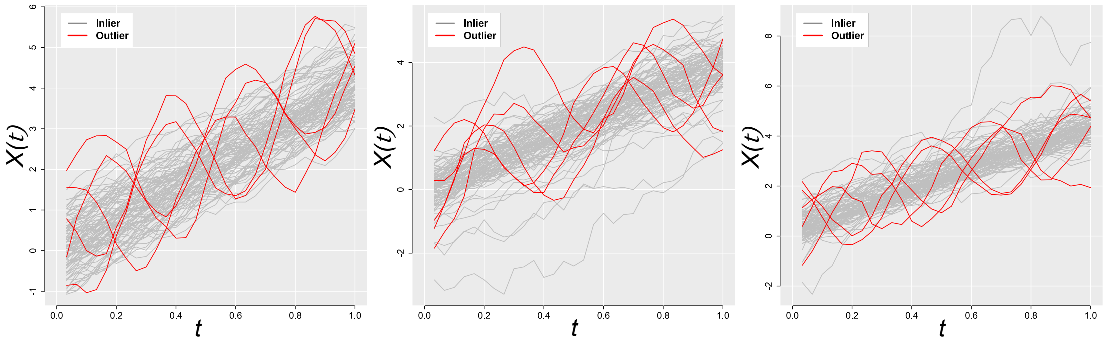

(a) (b) (c)

Figure S1 Data generated by Model 4, Model 5 and Model 6 in Example 2. (a) Model 4. (b) Model 5. (c) Model 6.

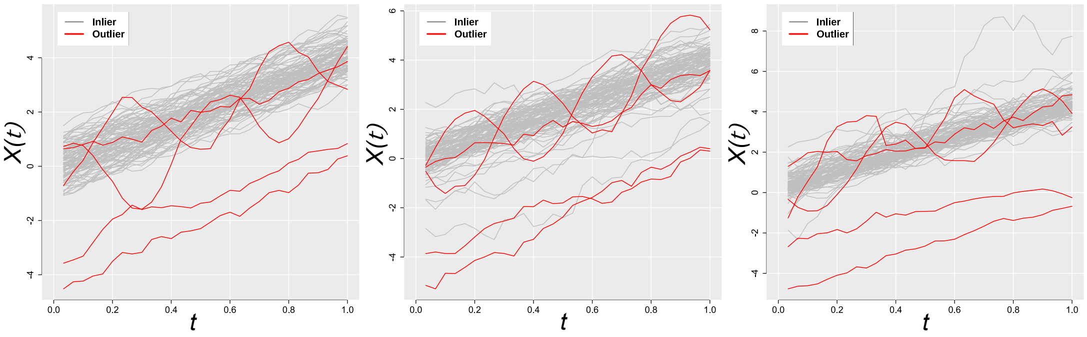

(a) (b) (c)

Figure S2 Data sample plots of Model 9, Model 10 and Model 11 Example 4. (a) Model 9. (b) Model 10. (c) Model 11.

Table S2. Example 2 result (Standard Deviations in Parentheses, $n = 50$)

		c=2%		c=4%		c=10%	
		p_c	p_f	p_c	p_f	p_c	p_f
Model 4	W1_1	100.0%	1.6%	100.0%	1.1%	100.0%	0.9%
		(0.0000)	(0.0206)	(0.0000)	(0.0173)	(0.0000)	(0.0158)
	W1_2	100.0%	1.8%	100.0%	1.5%	100.0%	1.1%
		(0.0000)	(0.0218)	(0.0000)	(0.0198)	(0.0000)	(0.0177)
	W2	100.0%	2.3%	100.0%	2.6%	100.0%	2.2%
		(0.0000)	(0.0263)	(0.0000)	(0.026)	(0.0000)	(0.0242)
	W3	45.0%	0.3%	100.0%	0.2%	33.8%	0.2%
		(0.5000)	(0.0104)	(0.0000)	(0.0079)	(0.2456)	(0.0067)
	W4	21.0%	8.8%	100.0%	8.9%	38.4%	8.6%
		(0.4093)	(0.0499)	(0.0000)	(0.0497)	(0.2676)	(0.0609)
	W5	100.0%	7.5%	100.0%	6.9%	100.0%	5.2%
		(0.0000)	(0.0378)	(0.0000)	(0.0357)	(0.0000)	(0.0333)
Model 5	W1_1	100.0%	5.1%	99.5%	4.7%	98.4%	3.4%
		(0.0000)	(0.0349)	(0.0500)	(0.0338)	(0.0677)	(0.0318)
	W1_2	100.0%	5.5%	100.0%	5.2%	99.0%	3.9%
		(0.0000)	(0.0365)	(0.0000)	(0.0351)	(0.0438)	(0.0323)
	W2	100.0%	6.8%	100.0%	6.5%	100.0%	5.9%
		(0.0000)	(0.0417)	(0.0000)	(0.0408)	(0.0000)	(0.0425)
	W3	36.0%	4.2%	33.5%	4.0%	31.0%	3.5%
		(0.4824)	(0.0358)	(0.3337)	(0.0351)	(0.2564)	(0.0360)
	W4	26.0%	14.8%	27.5%	15.2%	31.8%	13.5%
		(0.4408)	(0.0512)	(0.3435)	(0.0585)	(0.2528)	(0.0504)
	W5	100.0%	11.7%	100.0%	10.9%	98.8%	9.0%
		(0.0000)	(0.0397)	(0.0000)	(0.0385)	(0.0556)	(0.0393)
Model 6	W1_1	100.0%	7.3%	100.0%	6.8%	100.0%	5.4%
		(0.0000)	(0.0309)	(0.0000)	(0.0316)	(0.0000)	(0.0288)
	W1_2	100.0%	8.1%	100.0%	7.5%	100.0%	5.9%
		(0.0000)	(0.0330)	(0.0000)	(0.0325)	(0.0000)	(0.0311)
	W2	100.0%	7.8%	100.0%	7.4%	100.0%	6.2%
		(0.0000)	(0.0424)	(0.0000)	(0.0381)	(0.0000)	(0.0352)
	W3	100.0%	3.7%	50.0%	3.5%	44.6%	3.3%
		(0.0000)	(0.0282)	(0.4143)	(0.0258)	(0.2992)	(0.0247)
	W4	100.0%	11.1%	31.0%	10.4%	31.0%	9.4%
		(0.0000)	(0.0443)	(0.3316)	(0.0417)	(0.2367)	(0.0416)
	W5	100.0%	12.2%	100.0%	11.2%	99.4%	8.3%
		(0.0000)	(0.0390)	(0.0000)	(0.0372)	(0.0343)	(0.0339)

Table S3. Example 3 result (Standard Deviations in Parentheses, $n = 50$)

		c=2%		c=4%		c=10%	
		$\boldsymbol{p_c}$	$\boldsymbol{p_f}$	$\boldsymbol{p_c}$	$\boldsymbol{p_f}$	$\boldsymbol{p_c}$	$\boldsymbol{p_f}$
Model 0	W1_1	91.0% (0.2876)	1.9% (0.0243)	87.0% (0.2204)	1.9% (0.0248)	85.2% (0.1648)	1.7% (0.0232)
	W1_2	85.0% (0.3588)	2.0% (0.0233)	77.0% (0.3289)	1.7% (0.0198)	67.8% (0.2980)	1.2% (0.0176)
	W2	38.0% (0.4878)	2.3% (0.0251)	37.0% (0.3667)	2.2% (0.0234)	28.2% (0.2293)	2.0% (0.0231)
	W3	21.0% (0.4094)	0.4% (0.0117)	21.5% (0.2776)	0.2% (0.0079)	20.8% (0.2028)	0.6% (0.0187)
	W4	12.0% (0.3266)	9.0% (0.0495)	15.0% (0.2706)	9.2% (0.0516)	12.8% (0.1621)	9.2% (0.0515)
	W5	92.0% (0.2730)	7.5% (0.0360)	85.0% (0.2610)	7.3% (0.0361)	79.2% (0.2160)	5.7% (0.0376)
Model 7	W1_1	52.0% (0.5021)	5.2% (0.0338)	53.0% (0.4071)	4.8% (0.0358)	38.6% (0.3269)	4.4% (0.0362)
	W1_2	55.0% (0.5000)	5.6% (0.0374)	53.5% (0.4098)	5.5% (0.0387)	41.2% (0.3213)	4.6% (0.0387)
	W2	25.0% (0.4352)	6.9% (0.0420)	26.5% (0.3367)	6.4% (0.0438)	21.4% (0.2206)	5.6% (0.0435)
	W3	27.0% (0.4462)	4.4% (0.0360)	23.5% (0.3052)	4.2% (0.0365)	21.6% (0.1857)	4.3% (0.0415)
	W4	27.0% (0.4462)	15.4% (0.0532)	23.0% (0.2879)	15.1% (0.0538)	18.6% (0.1688)	15.0% (0.0561)
	W5	65.0% (0.4790)	12.0% (0.0436)	55.5% (0.3950)	11.5% (0.0385)	47.2% (0.2720)	9.9% (0.0421)
Model 8	W1_1	73.0% (0.4461)	7.6% (0.0323)	72.0% (0.3357)	7.2% (0.0311)	65.9% (0.2905)	6.5% (0.0340)
	W1_2	77.0% (0.4229)	8.2% (0.0330)	73.0% (0.3365)	7.7% (0.0311)	66.3% (0.2924)	7.0% (0.0343)
	W2	31.0% (0.4648)	7.6% (0.0387)	31.0% (0.3239)	7.1% (0.0359)	24.7% (0.2385)	6.0% (0.0363)
	W3	26.0% (0.4408)	3.8% (0.0287)	34.0% (0.3324)	3.6% (0.0276)	27.7% (0.2019)	3.4% (0.0248)
	W4	14.0% (0.3487)	11.4% (0.0473)	14.0% (0.2570)	10.9% (0.0400)	14.6% (0.1597)	11.0% (0.0432)
	W5	64.0% (0.4820)	12.4% (0.0363)	62.5% (0.3510)	11.9% (0.0376)	48.6% (0.2640)	9.7% (0.0389)

Table S4. Example 4 result (Standard Deviations in Parentheses, $n = 50$)

		c=1%		c=5%		c=10%	
		p_c	p_f	p_c	p_f	p_c	p_f
Model 9	W1_1	90.0% (0.3015)	1.3% (0.0169)	94.5% (0.1572)	1.1% (0.0146)	73.0% (0.1283)	0.9% (0.0141)
	W1_2	85.0% (0.3588)	1.4% (0.0171)	93.0% (0.1743)	1.3% (0.0161)	72.6% (0.1353)	1.1% (0.0171)
	W2	94.0% (0.2387)	2.1% (0.0325)	96.0% (0.1363)	1.7% (0.0273)	76.0% (0.0943)	1.5% (0.0233)
	W3	100.0% (0.0000)	0.3% (0.0097)	68.0% (0.2412)	0.2% (0.0088)	53.4% (0.1422)	0.2% (0.0102)
	W4	100.0% (0.0000)	8.1% (0.0459)	63.0% (0.2204)	7.6% (0.0449)	56.2% (0.1600)	7.3% (0.0437)
	W5	100.0% (0.0000)	7.8% (0.0367)	100.0% (0.0000)	7.4% (0.0376)	80.2% (0.0200)	6.6% (0.0383)
Model 10	W1_1	98.0% (0.1400)	5.1% (0.0365)	71.0% (0.2580)	4.4% (0.0322)	51.0% (0.1514)	3.9% (0.0346)
	W1_2	73.3% (0.4447)	5.4% (0.0343)	84.0% (0.2344)	5.1% (0.0348)	68.0% (0.1681)	4.2% (0.0346)
	W2	88.1% (0.3251)	6.4% (0.0399)	93.5% (0.1689)	6.6% (0.0430)	73.0% (0.1541)	6.3% (0.0447)
	W3	99.0% (0.0995)	4.1% (0.0354)	70.5% (0.2472)	4.1% (0.0367)	54.8% (0.1520)	3.6% (0.0355)
	W4	100.0% (0.0000)	14.5% (0.0497)	65.0% (0.2303)	14.8% (0.0538)	56.0% (0.1477)	14.0% (0.0589)
	W5	100.0% (0.0000)	12.6% (0.0415)	100.0% (0.0000)	11.4% (0.0398)	81.6% (0.0735)	10.2% (0.0408)
Model 11	W1_1	100.0% (0.0000)	7.5% (0.0364)	100.0% (0.0000)	6.9% (0.0338)	80.8% (0.0393)	6.0% (0.0340)
	W1_2	100.0% (0.0000)	8.0% (0.0366)	100.0% (0.0000)	7.5% (0.0355)	80.8% (0.0393)	6.5% (0.0353)
	W2	100.0% (0.0000)	6.5% (0.0360)	99.5% (0.0500)	6.3% (0.0361)	80.8% (0.0393)	6.0% (0.0360)
	W3	99.0% (0.1000)	3.7% (0.0308)	74.5% (0.2611)	3.4% (0.0288)	60.8% (0.1703)	3.7% (0.0291)
	W4	100.0% (0.0000)	11.4% (0.0517)	65.0% (0.2303)	10.9% (0.0477)	52.8% (0.1407)	11.0% (0.0465)
	W5	100.0% (0.0000)	13.0% (0.0395)	100.0% (0.0000)	12.3% (0.0373)	81.6% (0.0545)	11.0% (0.0368)

Table S5. Example 4 result on precision and F1 score

		c=1%		c=5%		c=10%	
		**Precision**	F_1	**Precision**	F_1	**Precision**	F_1
Model 9	W1_1	0.23	0.37	0.59	0.67	0.85	0.89
	W1_2	0.20	0.33	0.57	0.65	0.84	0.88
	W2	0.25	0.38	0.61	0.68	0.84	0.89
	W3	0.83	0.91	0.92	0.60	0.98	0.70
	W4	0.12	0.21	0.26	0.35	0.52	0.57
	W5	0.11	0.20	0.37	0.51	0.66	0.80
Model 10	W1_1	0.12	0.22	0.37	0.47	0.63	0.65
	W1_2	0.10	0.17	0.35	0.48	0.63	0.73
	W2	0.11	0.19	0.36	0.49	0.61	0.73
	W3	0.34	0.51	0.58	0.52	0.82	0.66
	W4	0.07	0.13	0.18	0.27	0.38	0.48
	W5	0.07	0.14	0.27	0.41	0.53	0.69
Model 11	W1_1	0.09	0.17	0.31	0.45	0.58	0.74
	W1_2	0.09	0.16	0.31	0.44	0.58	0.73
	W2	0.11	0.19	0.35	0.49	0.60	0.75
	W3	0.33	0.50	0.58	0.53	0.77	0.68
	W4	0.09	0.16	0.20	0.28	0.39	0.48
	W5	0.07	0.14	0.27	0.41	0.52	0.68

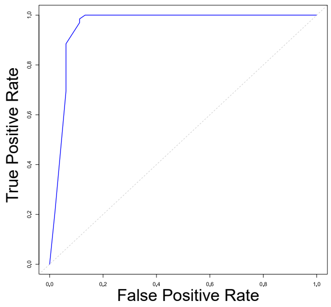

(a) W1_1

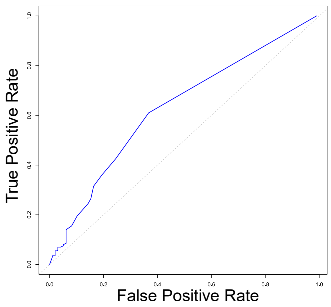

(b) W2

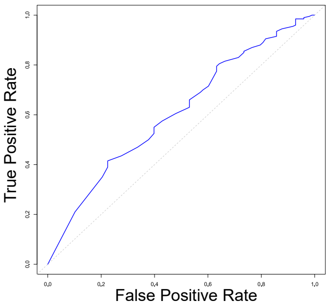

(c) W3

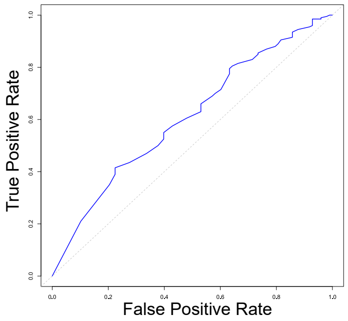

(d) W4

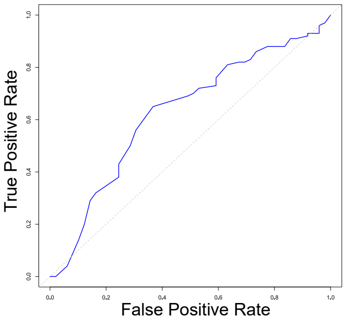

(e) W5

Figure S3. Average ROC curves of Model 8. (a) W1_1. (b) W2. (c) W3. (d) W4 (e) W5.

2. Results on real data analysis

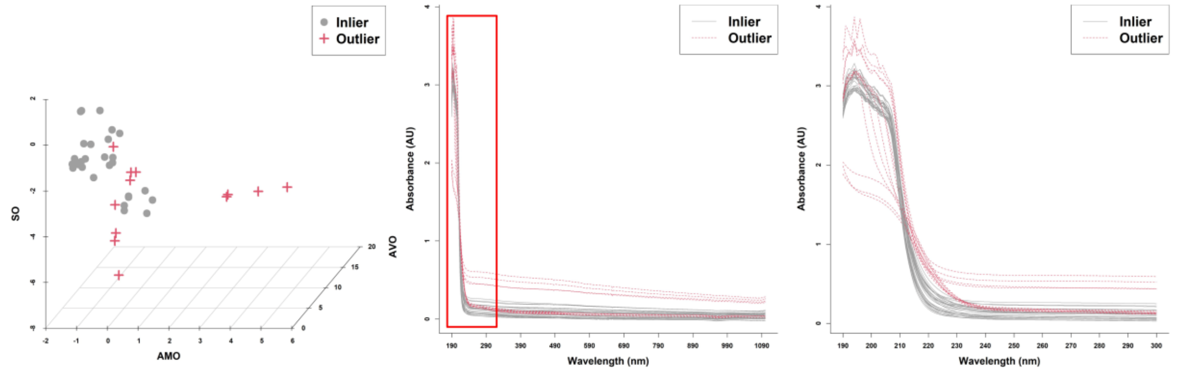

(a) (b) (c)

Figure S4. Outlier detection result of adjusted outlyingness detection method. (a) 3D plot of $(AVO, AMO, SO)^{\mathrm{T}}$. (b) Spectral data outlier detection result. (c) Wavelength range from 190 nm to 300 nm.

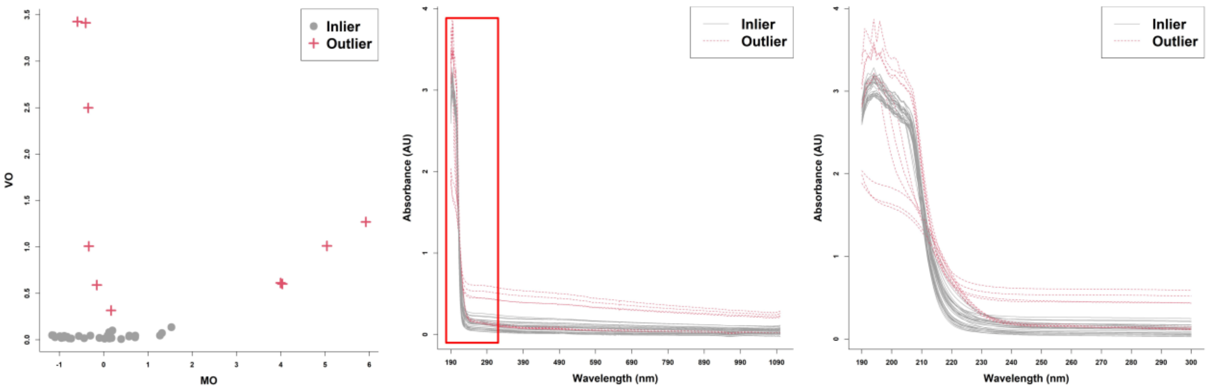

(a) (b) (c)

Figure S5. Outlier detection result of outlyingness detection method. (a) 2D plot of $(MO, VO)^{\mathrm{T}}$. (b) Spectral data outlier detection result. (c) Wavelength range from 190 nm to 300 nm.

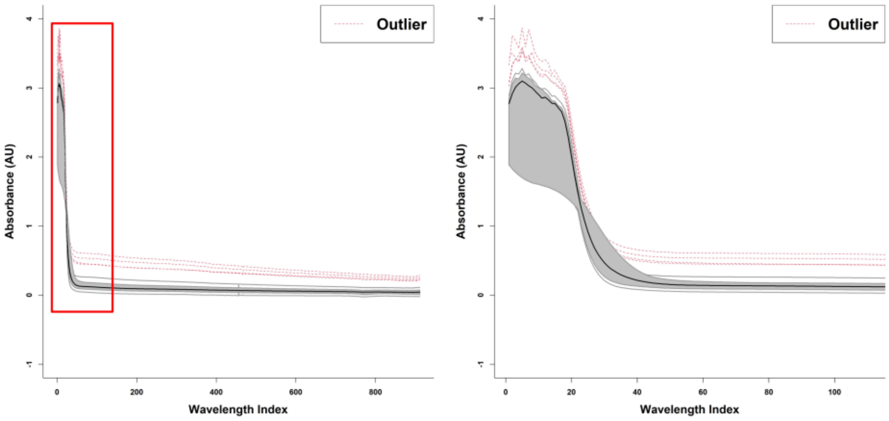

(a) (b)

Figure S6. Outlier detection result of functional boxplot method. (a) Spectral data outlier detection result. (b) Wavelength index from 0 to 110

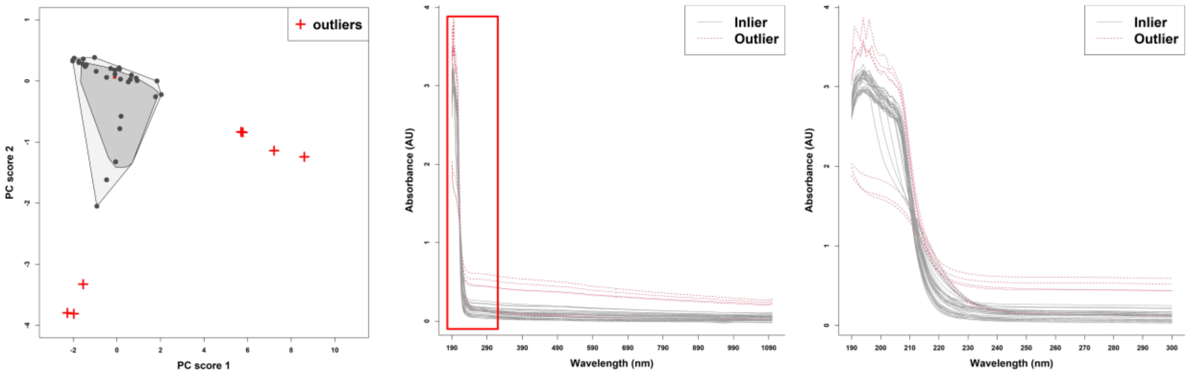

(a) (b) (c)

Figure S7. Outlier detection result of functional bagplot method. (a) PCA scatter plot with outliers marked in red. (b) Spectral data outlier detection result. (c) Wavelength range from 190 nm to 300 nm.

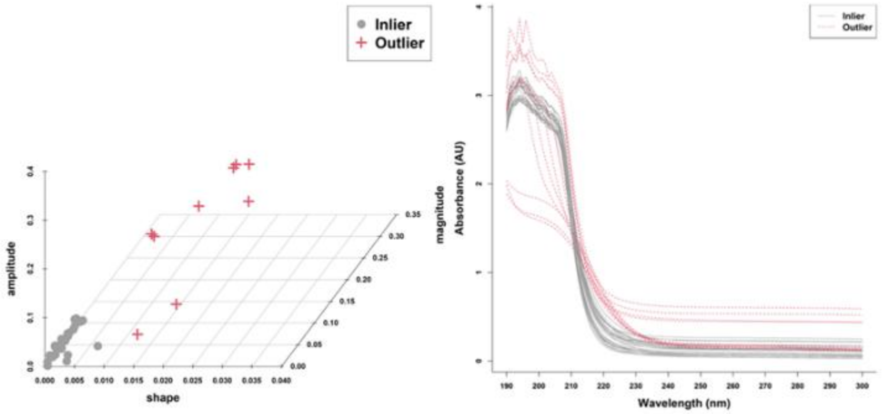

(a) (b)

Figure S8. Outlier detection result of FAST-MUOD method. (a) Scatter plot with outliers marked in red. (b) Spectral data outlier detection result. (c) Wavelength range from 190 nm to 300 nm.

3. Significance Test

We conducted the Friedman test and Wilcoxon test under 0.05 significant level to show the statistical significance of on p_c and p_f of our proposed method in the simulation study. Table S6 records the p-value of each Friedman test under different outlier proportion with results recorded in simulation study accordingly. Tables S7-S9 shows the Wilcoxon test p-value under 0.05 significant level comparing between each two methods.

Table S6. Friedman test p-value on p_c and p_f

	$c = 1\%$	$c = 5\%$	$c = 10\%$
p_c	0.005 *	9.866e-05 *	0.002 *
p_f	4.236e-10 *	3.903e-10 *	2.741e-10 *

*：Significant under 0.05 level

Table S7. Wilcoxon test p-value ($c = 1\%$)

	p_c	p_f
W1_1 vs W1_2	0.584	0.037 *
W1_1 vs W2	0.036 *	0.029 *
W1_1 vs W3	0.017 *	0.003 *
W1_1 vs W4	0.035 *	0.003 *
W1_1 vs W5	0.136	0.003 *

*：Significant under 0.05 level

Table S8. Wilcoxon test p-value ($c = 5\%$)

	p_c	p_f
W1_1 vs W1_2	0.611	0.045 *
W1_1 vs W2	0.272	0.367
W1_1 vs W3	0.008 *	0.003 *
W1_1 vs W4	0.008 *	0.003 *
W1_1 vs W5	0.800	0.003 *

*：Significant under 0.05 level

Table S9. Wilcoxon test p-value ($c = 10\%$)

	p_c	p_f
W1_1 vs W1_2	0.575	0.036 *
W1_1 vs W2	0.554	0.7241
W1_1 vs W3	0.009 *	0.003 *
W1_1 vs W4	0.011 *	0.003 *
W1_1 vs W5	0.813	0.003 *

*：Significant under 0.05 level

Based on Tables S6-S9, the Friedman test and Wilcoxon test demonstrate the superiority of our method. The improvements in p_c and p_f o are statistically significant.

4. Real Data Application

Population Data

It was found that most of these regions underwent drastic political and economic system ransformations in the late 1980s to early 1990s. This historical context is clearly linked to the turning point in their demographic trends, which is statistically manifested as an anomalous "rise-then-fall" pattern. Therefore, in the main text, we refer to countries with an overall upward trend as normal samples, while those exhibiting a trend shift around 1990 are termed anomalous samples. See the following Table S10.

Table S10. Anomaly Samples (Countries and Regions)

Albania	Estonia	North Macedonia
Armenia	Georgia	Republic of Moldova
Belarus	Hungary	Romania
Bosnia and Herzegovina	Kosovo (under UNSC res. 1244)	Russian Federation
Bulgaria	Latvia	Serbia
Croatia	Lithuania	Ukraine
